# What's in a tide pool? Just as much food web network complexity as in large open ecosystems

**DOI:** 10.1371/journal.pone.0200066

**Published:** 2018-07-05

**Authors:** Vanessa Mendonça, Carolina Madeira, Marta Dias, Fanny Vermandele, Philippe Archambault, Awantha Dissanayake, João Canning-Clode, Augusto A. V. Flores, Ana Silva, Catarina Vinagre

**Affiliations:** 1 MARE–Marine and Environmental Sciences Centre, Faculdade de Ciências, Universidade de Lisboa, Campo Grande, Lisboa, Portugal; 2 Université du Québec à Rimouski, Département de Biologie, Chimie et Géographie, Rimouski, Canada; 3 Département de biologie, Takuvik, Québec-Océan, Université Laval, Québec, Canada; 4 School of Biological Sciences, Plymouth University, Plymouth, United Kingdom; 5 MARE–Marine and Environmental Sciences Centre, Quinta do Lorde Marina, Sítio da Piedade, Caniçal, Madeira Island, Portugal; 6 Centre of IMAR of the University of the Azores, Department of Oceanography and Fisheries, Rua Prof. Dr. Frederico Machado, Horta, Azores, Portugal; 7 Smithsonian Environmental Research Center, Edgewater, MD, United States of America; 8 Centro de Biologia Marinha, Universidade de São Paulo, Rod. Manoel Hipólito do Rego, São Sebastião, SP, Brazil; 9 CERIS, Instituto Superior Técnico, Universidade de Lisboa, Lisboa, Portugal; University of Waikato, NEW ZEALAND

## Abstract

Understanding the fundamental laws that govern complex food web networks over large ecosystems presents high costs and oftentimes unsurmountable logistical challenges. This way, it is crucial to find smaller systems that can be used as proxy food webs. Intertidal rock pool environments harbour particularly high biodiversity over small areas. This study aimed to analyse their food web networks to investigate their potential as proxies of larger ecosystems for food web networks research. Highly resolved food webs were compiled for 116 intertidal rock pools from cold, temperate, subtropical and tropical regions, to ensure a wide representation of environmental variability. The network properties of these food webs were compared to that of estuaries, lakes and rivers, as well as marine and terrestrial ecosystems (46 previously published complex food webs). The intertidal rock pool food webs analysed presented properties that were in the same range as the previously published food webs. The niche model predictive success was remarkably high (73–88%) and similar to that previously found for much larger marine and terrestrial food webs. By using a large-scale sampling effort covering 116 intertidal rock pools in several biogeographic regions, this study showed, for the first time, that intertidal rock pools encompass food webs that share fundamental organizational characteristics with food webs from markedly different, larger, open and abiotically stable ecosystems. As small, self-contained habitats, intertidal rock pools are particularly tractable systems and therefore a large number of food webs can be examined with relatively low sampling effort. This study shows, for the first time that they can be useful models for the understanding of universal processes that regulate the complex network organization of food webs, which are harder or impossible to investigate in larger, open ecosystems, due to high costs and logistical difficulties.

## Introduction

Comparative analysis of food webs from different habitats has revealed generalities in the subjacent network structure of trophic interactions. Estuarine, marine, stream, lake, and terrestrial ecosystems all seem to share similar general properties of complex food web network structure [1–5].

Initially, food web networks from marine ecosystems were thought to be different from those of other ecosystems [6, 7], in that they presented higher average links per species, chain lengths and connectivity than non-marine ecosystems. Yet, [3] showed that those differences were due to different scales used in the analyses. Food web network properties are scale-dependent, changing as diversity and complexity change [8, 9] and thus direct comparisons can be misleading. In fact, [3] demonstrated that marine food webs are not different from non-marine food webs, by comparing their fit to the theoretical niche food web model [1]. The niche model incorporates scale-dependence, hence allowing the comparison of food webs with different diversity and complexity.

Intertidal rocky shores are among the most intensely studied ecosystems in the world. They provide a natural laboratory where abiotic stress, biotic interactions and biological patterns can be easily examined [10–13]. However, intertidal rock pools have received much less attention than the surrounding emergent intertidal bedrock, and thus much less is known about their community dynamics [14–16]. This is mainly due to their high structural variability, which makes proper replication of sampling units very challenging [14, 17].

Intertidal rock pools are isolated mesocosms of permanently immersed habitat, surrounded by intermittently emerged rock surfaces. Environmental conditions in these pools are much less harsh than in the surrounding environment (e.g. high temperature amplitudes, desiccation stress). They allow many organisms to extend their upper vertical limits [18–22], provide refuge [22–24], feeding habitats [25, 26] and nursery grounds [26–28] for many marine species. It is also generally acknowledged that the use of intertidal rock pools during early ontogeny (e.g. fish, shrimp) is likely to enhance growth, fitness and the survival chances of the individuals that use them [29–31].

Intertidal rock pools are ubiquitous features of rocky shores in many parts of the world and can harbour rich biodiversity [16]. Studies have been carried out focusing on their community structure [12, 15, 17], and the roles of herbivory on community structure [32–34] competition [34, 35] predation [36–38] and recruitment [35]. However, to the best of our knowledge, the network structure of food webs that occurs in intertidal rock pools remains unknown. The issue of whether complex food web networks can develop in such small and abiotically variable environments is yet to be uncovered. Given their accessibility and easy manipulation, these natural mesocosms could be useful models for the understanding of universal processes that regulate the complex organization of food webs, which are harder or impossible to investigate in open ecosystems.

The aim of the present study is to analyse, for the first time, the complex network structure of food webs occurring in intertidal rock pools and compare it to the ones of other habitats, by estimating their network properties and fit to the theoretical niche food web model (the network from each pool was compared to 1000 automatically generated food web networks). For this purpose, a significant number (n = 116) of intertidal rock pools were investigated in different biogeographic regions of the world, to encompass a wide range of potential variability, and compared to 46 other previously published food webs, from estuarine, marine, stream, lake, and terrestrial ecosystems. By doing this, we aim to investigate the potential use of rock pools as proxies of larger ecosystems for food web networks research.

## Results

The data assembled for the intertidal rock pool food webs resulted in lists of 11 to 68 taxa per pool. These taxa corresponded to lists of 7 to 52 trophic species per pool. Some biological compartments needed to be aggregated due to low definition of predator’s diet, which impeded the construction of prey-predator links at the taxonomic species level. This was particularly evident for phytoplankton, zooplankton, oligochaeta and polychaeta.

The comparison of the range of food web network properties among intertidal rock pools and other ecosystems, reported in previous works (Table 1), showed that intertidal rock pools’ properties are generally within the range estimated for other ecosystems.

**Table 1:** Ranges of commonly reported structural food-web properties for food webs from rock intertidal pools and a variety of other ecosystem types.

Ecosystem	N	S	C	L/S	T	I	B	Can	Omn	TL	Chain	Path	Source
Rock tide pools (all pools)	116	**7**–52	0.11–**0.39**	1.6–7.0	0–46	**14**–88	7–43	14–60	43–84	1.68–2.5	1.57–2.00	1.27–1.97	Present work
Rock tide pools 48°N, Gulf St. Lawrence–Canada	28	**7****–****15**	0.19–0.29	1.6–4.0	7–43	**14**–71	20–43	14–50	43–73	1.68–2.16	1.57–1.80	1.48–1.78	
Rock tide pools 50°N, UK	8	**15**–25	0.24–0.32	5.0–7.0	0–10	75–88	12–20	33–60	65–84	2.17–2.30	1.80–1.88	1.44–1.56	
Rock tide pools 38°N, Portugal-west coast	32	**15**–52	0.11–0.29	4.0–7.0	0–20	65–87	7–21	20–38	53–84	1.99–2.36	1.79–1.96	1.47–1.97	
Rock tide pools 32°N, Portugal-Madeira	14	**11****–****24**	0.20–**0.39**	3.0–5.0	0–10	62–79	16–27	31–58	62–79	2.05–2.35	1.73–1.84	1.27–1.72	
Rock tide pools 23°S, Brazil-SP	18	**10**–26	0.13–0.24	3.0–7.0	0–19	55–88	15–30	27–47	57–83	2.00–2.50	1.73–1.88	1.38–1.64	
Rock tide pools 3°S, Brazil-CE	16	**11**–26	0.19–0.33	2.0–3.0	8–46	27–77	12–27	11–33	64–84	1.90–2.41	1.7–2.0	1.59–1.93	
Seagrass beds	16	53–68	0.17–0.23	11.4–12.9	13–18	58–65	21–26	13–19	70–75	1.8–2.0	1.9–2.0	2.0–2.3	[39]
Marine	4	29–245	0.05–0.24	7.0–17.8	0–4	93–98	2–7	4–42	76–87	2.9–3.2	6.4–15.3	1.6–1.9	[3, 7, 40, 41]
Estuarine	12	48–117	0.03–0.14	2.0–10.1	7–52	31–86	4–20	1–24	53–84	2.4–2.9	4.0–6.6	2.0–2.7	[5, 42–47]
Lake/pond	5	25–172	0.12–0.32	4.3–25.1	0–9	66–92	4–32	12–32	38–60	2–2.7	4.0–10.7	1.3–1.9	[3, 48–50]
Stream	5	31–109	0.07–0.13	3.7–7.6	6–25	22–86	7–56	1–2	6–10	1.5–3.4	3.1–3.2	2.3–2.3	[51, 52]
Terrestrial	4	29–155	0.03–0.31	1.6–9.0	0–31	56–90	13–18	0–66	21–76	2.4–3	3.2–8.4	1.4–3.7	[53–56]

S = number of trophic species, C = connectance, L/S = links per species, T = % top species, I = % intermediate species, B = % basal species, Can = % cannibalistic species, Omn = % omnivorous species, TL = mean trophic level, Chain = mean number of links in every possible food chain or sequence of links connecting top species to basal species, Path = characteristic path length (ranges that do not totally overlap with those of other non-marine ecosystems are presented in bold; ranges that do not totally overlap with those of other marine ecosystems are underlined).

**Fig 1:**
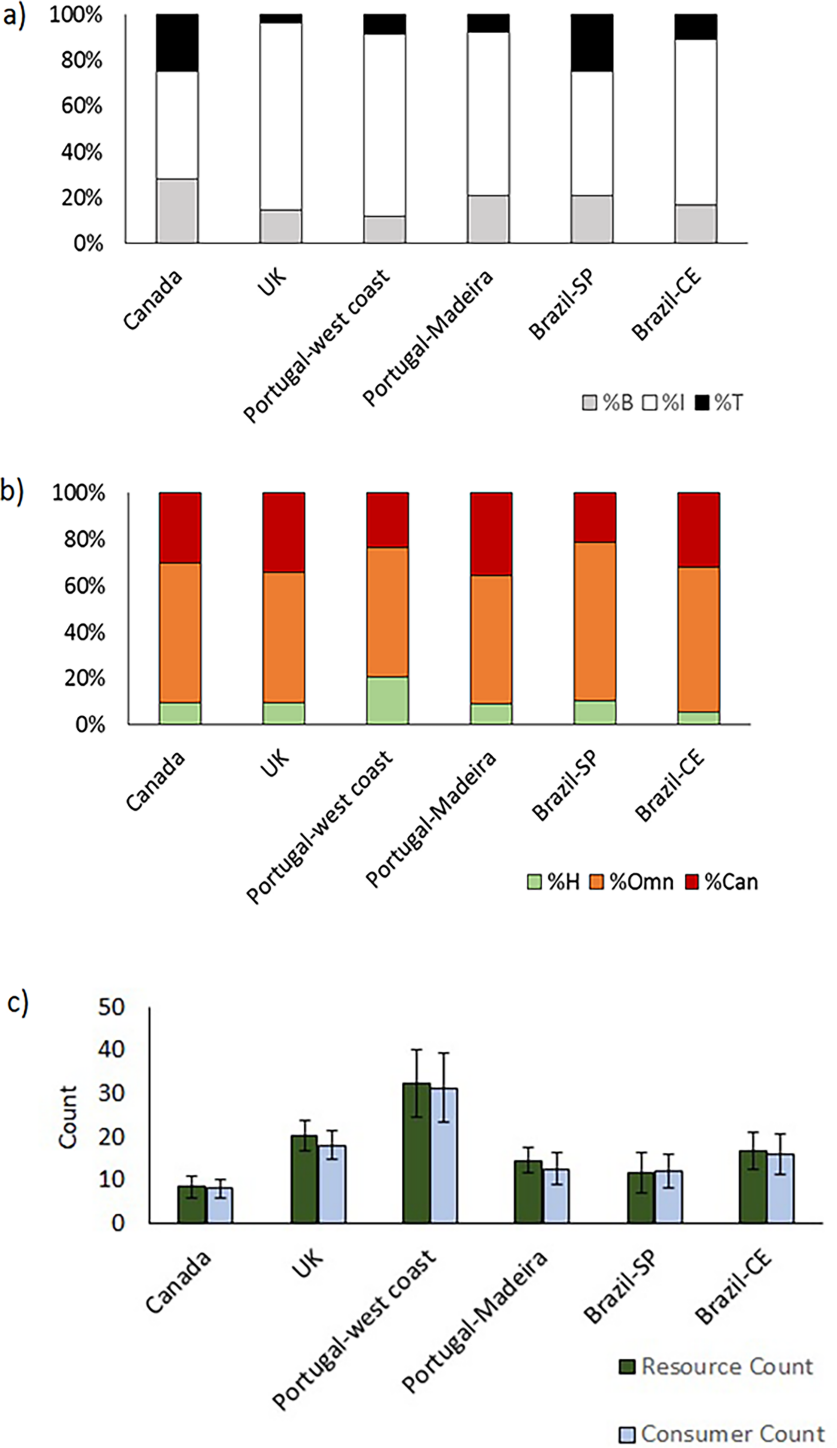
Variation in the basic properties of the food web networks of the rock intertidal pools. (a) percentage of top species (%T), percentage of intermediate species (%I) and percentage of basal species (%B); (b), percentage of herbivore species (%H), percentage of cannibal species (%Can) and percentage of omnivore species (%Omn) and (c) Resource and Consumer counts (mean values for all pools).

The number of food web structural networks reported in the present work, n = 116, is remarkably higher than those previously reported for all other ecosystems combined, n = 46 (Table 1). The number of trophic species (S) observed in intertidal rock pools was considerably lower, 7–52, than that reported for all other ecosystems, 25–245, however such number of trophic species refers to much smaller areas (Table 1). Connectance in some intertidal rock pools was higher than that reported for other ecosystems (Table 1). Links per species (L/S) was lower, 1.6–7.0, than previously observed for marine systems, 7.0–17.8, but within the range reported for most non-marine ecosystems, 2.0–25.1. The percentage of intermediate species (I) showed lower bottom values, 14%, when compared to all other systems, 22–98%, due to the very low values reported for some pools in Canada. The highest values found for the percentage of basal species (B) and cannibal species (Can), 43% and 60%, respectively, were higher than those found for other marine systems, 26% and 42% respectively, however B was within the previously reported range for non-marine ecosystems, 4–56%, unlike Can which was not, since the percentage of Can in non-marine systems ranges between 1 and 32% (Table 1, Fig 1). The lowest values found for the percentage of omnivorous species (Omn), 43%, were lower than reported for other marine systems, 70%, but within the ranges for non-marine ecosystems, 6–84% (Table 1, Fig 1). Mean trophic level (TL), mean number of links in every possible food chain (Chain) and mean shortest path length between species pairs (Path) were within the values reported for non-marine ecosystems, however their lower values were lower than those reported for other marine ecosystems (Table 1).

In addition, the taxa most frequently in the top 3 highest trophic level varied greatly among locations, however Polychaeta were among the top 3 in the UK, Brazil-SP and Brazil-CE, and the fish *Bathygobius soporator* in Brazil-SP and Brazil-CE (Table 2). The taxa that was most frequently in the top 3 highest trophic level was Oligochaeta in Canada, *Carcinus maenas* in the UK, Nematoda in Portugal-west coast, *Pachygrapsus transversus* in Portugal-Madeira, Polychaeta in Brazil-SP and in Brazil-CE (Table 2). The highest trophic level varied between 2.3 and 2.9 in Canada, between 2.8 and 2.9 in the UK, between 2.6 and 3.7 in Portugal-west coast, between 2.7 and 3.1 in Portugal-Madeira, between 2.6 and 3.6 in Brazil-SP and between 2.7 and 3.2 in Brazil-CE.

**Table 2:** Taxa most frequently in the top 3 of highest trophic level* and connectivity in the food webs analyzed.

	Taxa most frequently in the top 3 highest trophic level*	Number of webs where the taxa were in the top 3 highest trophic level*	Taxa most frequently in the top 3 highest connectivity	Number of webs where the taxa were in the top 3 highest connectivity	Total webs analysed
Canada					28
	Oligochaeta	17	Zooplankton	28	
	*Gammarus oceanicus* (Amphipoda)	13	Detritus	27	
	Zooplankton	13	Polychaeta	10	
UK					8
	*Carcinus maenas* (Crab)	5	Polychaeta	8	
	*Palaemon serratus* (Shrimp)	4	Detritus	6	
	Polychaeta	3	*Carcinus maenas* (Crab)	6	
Portugal-west coast					32
	Nematoda	20	Detritus	32	
	Nassaridae (Snail)	16	*Lypophrys pholis* (Fish)	25	
	*Anemonia sulcata* (Anemone)	16	*Anemonia sulcata* (Anemone)	23	
Portugal-Madeira					14
	*Pachygrapsus transversus* (Crab)	7	Detritus	11	
	*Palaemon elegans* (Shrimp)	5	Zooplankton	10	
	*Lypophrys pholis* (Fish)	5	*Pachygrapsus transversus* (Crab)	4	
Brazil-SP					18
	Polychaeta	12	Detritus	18	
	*Stramonita haemastoma* (Snail)	9	Zooplankton	18	
	*Bathygobius soporator* (Fish)		Polychaeta	7	
Brazil-CE					16
	Polychaeta	10	Polychaeta	16	
	*Bathygobius soporator* (Fish)	8	Zooplankton	10	
	*Pagurus* sp. (Crab)	7	Detritus	9	

* shortweighted

The taxa most frequently in the top 3 of highest connectivity were “detritus”, albeit not a taxon, this node was included in the webs and was always among the ones with highest connectivity, in all locations (Table 2). Zooplankton was also in this group in Canada, Portugal-Madeira, Brazil-SP and Brazil-CE (Table 2), and Polychaeta in Canada, the UK, Brazil-SP and Brazil-CE (Table 2). The highest values of connectivity varied between 1.5 and 2.0 in Canada, between 1.6 and 2.2 in the UK, between 1.1 and 3.9 in Portugal-west coast, between 1.4 and 2.1 in Portugal-Madeira, between 1.8 and 2.8 in Brazil-SP and between 1.7 and 2.5 in Brazil-CE.

No significant correlations were found for any of the food web properties and pool depth, area or height for the pools in Canada, Portugal-west coast, Portugal-Madeira and Brazil-CE. An important number of correlations (r^2^>0.5; p<0.05) were found for the pools in the UK, for area and L/S (r^2^ = 0.9), area and TL (r^2^ = 0.8), area and I (r^2^ = 0.7), area and resource count (r^2^ = 0.7), area and S (r^2^ = 0.6) and area and Omn (r^2^ = 0.5). In Brazil-SP a correlation between area and L/S (r^2^ = 0.5) was found.

**Fig 2:**
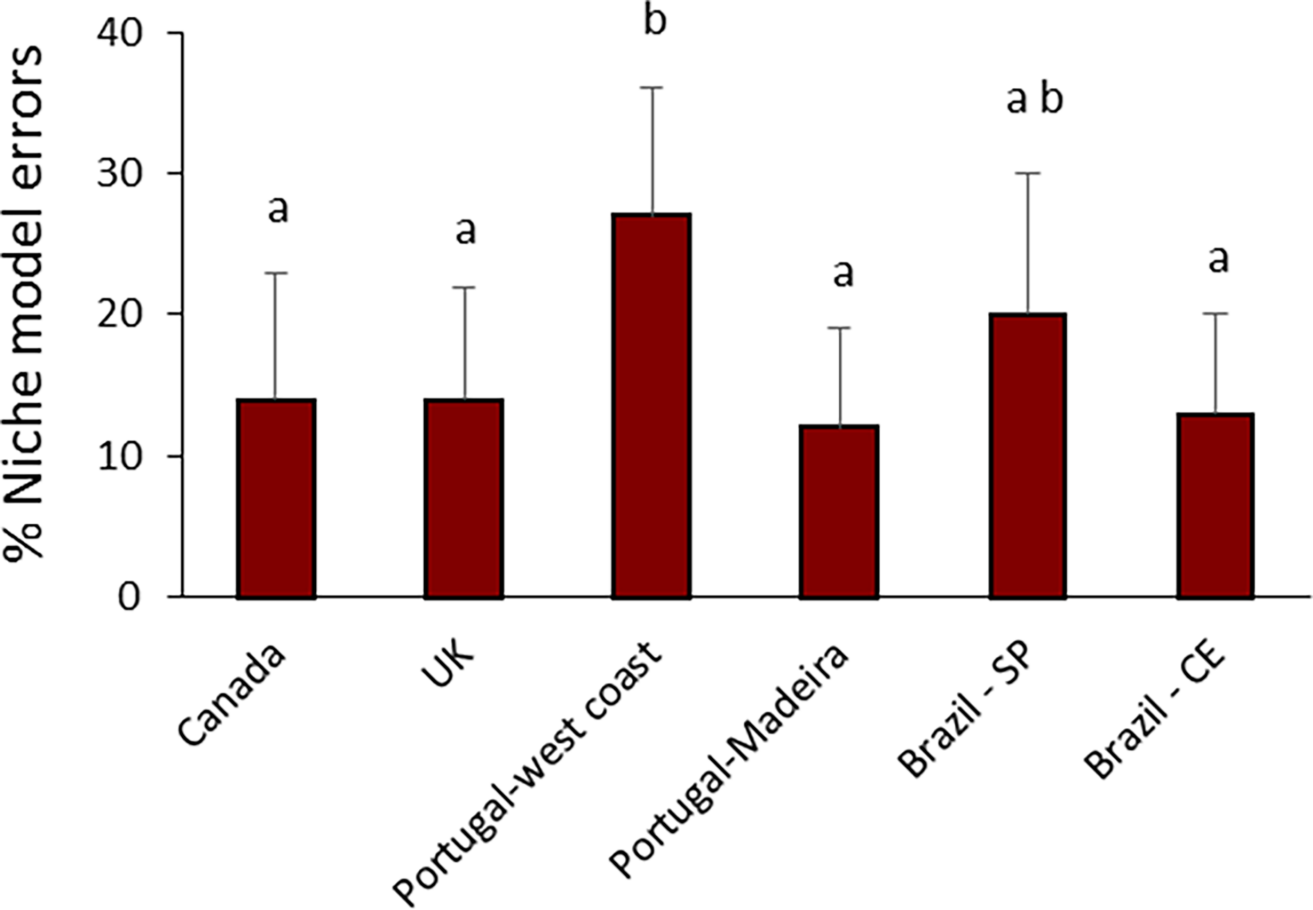
Percentage of niche model errors for 18 network structure properties (defined in Table 3) that are greater than |1|.

The percentage of niche model errors ranged between 12% (Portugal-Madeira) and 27% (Portugal-west coast) (Fig 2). The value was significantly higher for the intertidal rock pools surveyed in Portugal-west coast, in comparison to all other sites, with the exception of Brazil-SP (p<0.001; Fig 2).

**Fig 3:**
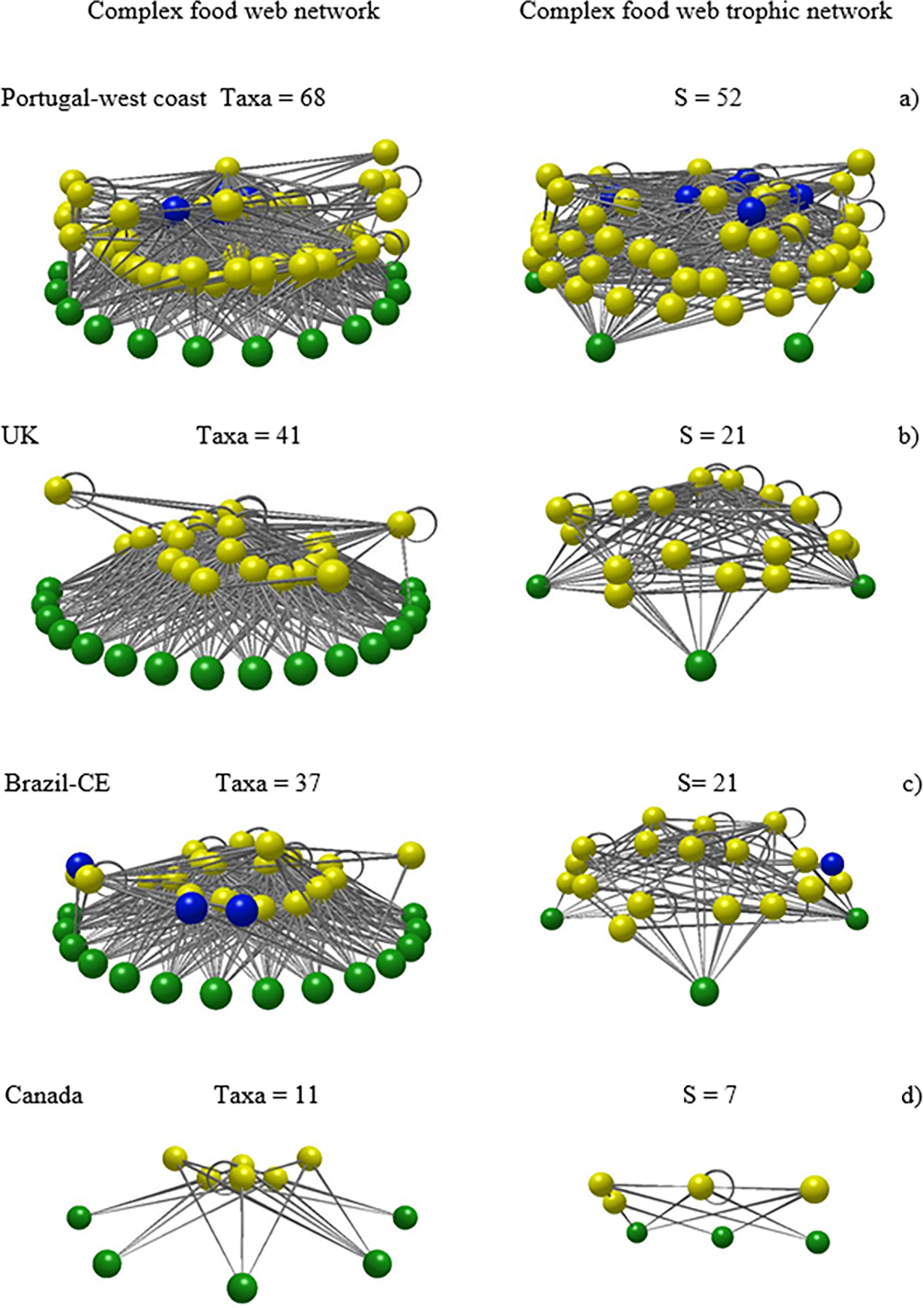
Network3D images of food web networks of selected rock intertidal pools. a–food web with the highest S, b and c–food webs with average S, d–food web in the lowest S. Green nodes = basal taxa; yellow nodes = invertebrates; blue nodes = vertebrates). On the left complex food web networks are depicted, on the right are the trophic species versions of the same food webs. Trophic species are groups of taxa whose members share the same set of predators and prey and are thus aggregated in single nodes.

The food webs produced by Network3D [57, 58] allowed the visual observation of the complexity of the food web networks analysed. Examples of food web networks that depict various levels of biodiversity were selected and shown in Fig 3, where the increasing complexity with increasing S is clear and easy to understand even by a non-specialized audience.

## Discussion

The clearest and most important conclusion of this work is that the food webs that occur in intertidal rock pools, albeit occupying very small areas, share the fundamental organizational structure of the food webs established over much larger open areas, such as marine areas, estuaries, rivers and terrestrial ecosystems, and thus can be useful for food web networks research. Nevertheless, there were some differences in the ranges of properties found for intertidal rock pools when compared to other ecosystems. Connectance is usually highest in food webs with a large proportion of intermediate and omnivorous species, like marine food webs [3]. Intertidal rock pools had high connectance but a relatively low proportion of intermediate and omnivorous species. This high connectance was, in the case of intertidal rock pools, related to a high proportion of top, basal and cannibalistic taxa. This difference may reside in the role that intertidal rock pools play as refuge and feeding areas for early stages of predatory species, mostly fish, crabs and shrimp [22, 26, 59]. Such early stages, despite their small size, are often already predators of smaller animals that also find refuge and food in intertidal rock pools. The high proportion of cannibalism is also explained by the occurrence of such early stages, given that often the same species larvae and various juvenile stages will find refuge in the same pool, and the larger individuals cannibalize the smaller conspecifics [22, 26, 59].

[3] hypothesized that the high proportion of intermediate, omnivorous and cannibal species found in marine food webs, when compared to non-marine food webs, could be related to: i) a resolution bias in marine datasets, that often present higher resolution for omnivorous commercial fish; ii) to a tendency to overlook cannibalistic relations in non-marine datasets or to iii) fundamental differences between the marine *versus* terrestrial food webs (e.g. widespread generality in marine systems based on gape size and the non-selectivity of filter feeding). These three hypotheses are discussed next.

Because intertidal species are well studied in the locations where the present study was conducted, some of the resolution biases that could be a problem in other marine studies were avoided, and that could be one of the reasons behind the lower proportion of intermediate and omnivorous species in intertidal rock pool food webs. The second hypothesis put forward by [3] that states that non-marine datasets overlook cannibalistic relations and have, therefore, a lower proportion of cannibalism could still hold true, although in the case of intertidal rock pools it can be argued the different sized life stages of predatory fish, crabs and shrimp find themselves together in a very small area, making cannibalism more unavoidable due to the impossibility of smaller life stages to escape. Pools are environments particularly prone to cannibalism [60–63] and the high proportion of cannibalism registered in the present study is probably an important particular characteristic of these food webs, as can be seen in the present study in the comparison between rock pools and large marine ecosystems (Table 1).

The results found for intertidal rock pools, albeit with some differences from other marine systems, also seem to support the third hypothesis put forward by [3], and previously proposed by [64], that marine systems have particular fundamental differences from non-marine systems, like feeding based on gape size and non-selective filter feeding by many primary and secondary consumers. The feeding based on gape size is the mechanism subjacent to the high proportion of cannibalism observed in intertidal rock pools [26]. This is well known for fish, prone to eat any conspecific given that its size fits its mouth opening [26].

The low proportion of basal species in marine food webs, found by [3], was considered a clear artifact of low resolution of basal taxa and of the consumer links directed towards them. [3] concluded that an improvement in resolution at the basal level would mitigate, but not erase, the high levels of intermediate, omnivorous and cannibal species in marine food webs. In the present study, due to a good knowledge and abundant literature on intertidal macroalgae it was possible to eliminate this artifact. This resulted in a proportion of basal species higher that previously reported for other marine systems, but which in fact should be closer to the real values for marine systems in general, once higher resolution of basal species is achieved for other marine environments as well. Given that the proportion of intermediate and omnivorous species was lower for intertidal rock pools than previously reported for marine systems, it can be concluded that the present study supports the hypothesis of [3], apart from cannibalism, which remained high despite the better resolution at the basal level.

The highest trophic level found for Portuguese intertidal rock pools, between 2.6 and 3.7, confirms a previous isotopic study conducted in the same area over the intertidal platform, including intertidal rock pools, which placed the highest TL of that food web at 3.3 [65]. The species that occupied the highest trophic level varied widely among pools and locations, encompassing oligochaeta, polychaeta, anemones, amphipods, gastropods, crustaceans and fish. This probably reflects not only the environmental characteristics of each location, but also individual pool characteristics, such as depth, available prey and algal cover.

The taxon most frequently in the top 3 of highest connectivity, over all locations, was “detritus”. Albeit not technically a taxon it was considered a node in the food web. Its high connectivity confirms the previous findings by e.g. [65, 66, 67], which noted that intertidal food webs rely heavily on detritus.

Although an important number of correlations were found between pool area and some network properties in the UK, such size-related trends were not observed in the other locations, suggesting that limitations for the size and complexity of trophic networks may vary across ecoregions, and highlighting the need for replicate sampling at different spatial scales for a better appraisal of general patterns. The niche model predictive success was remarkably high (73–88%) for intertidal rock pools. This predictive success rate is similar to the 79% previously found for 7 non-marine food webs [1] and the average 87% found for 3 marine food webs: the Benguela ecosystem off the coast of South Africa, a Caribbean coral reef ecosystem from the Puerto Rico—Virgin Islands shelf complex and a shelf ecosystem off the Northeast US [3].

The overlap in properties’ ranges between the rock pool food webs and previously published food webs (from a wide range of ecosystems) and their high fit to the niche model (among the highest ever published), lead to the conclusion that food web networks from rock pools have a great potential to be used as proxies of larger ecosystems for food web networks research. They are small and easy to sample, allowing greater replication and easy manipulation, two of the main challenges when dealing with large open systems. Although this approach would have some limitations, inherent to the use of one particular environment as proxy for vastly different environments and the uncertainty thereof, it would allow important advances resulting from the experimental manipulation of the web components and abiotic variables (e.g. algal coverage manipulation, predators’ exclusion, temperature, salinity) over many replicate food webs.

## Methods

### Sampling

The authors declare that the sampling followed the Portuguese, Brazilian, UK and Canadian legislation. Ethics committees in Portugal and Brazil specifically authorized this work. Authorization document 0421/000/000/2013 from the Portuguese authorities (DGAV) and 13.1.981.53.7 from the Brazilian authorities (CEUA, USP—Ribeirão Preto). The scientific permit delivered by Fisheries and Oceans Canada to Université du Québec à Rimouski, number 100003461, was used in Canada. No specific permissions were required for sampling in the field sites in the UK. The field work did not involve endangered or protected species in any of the areas.

**Fig 4:**
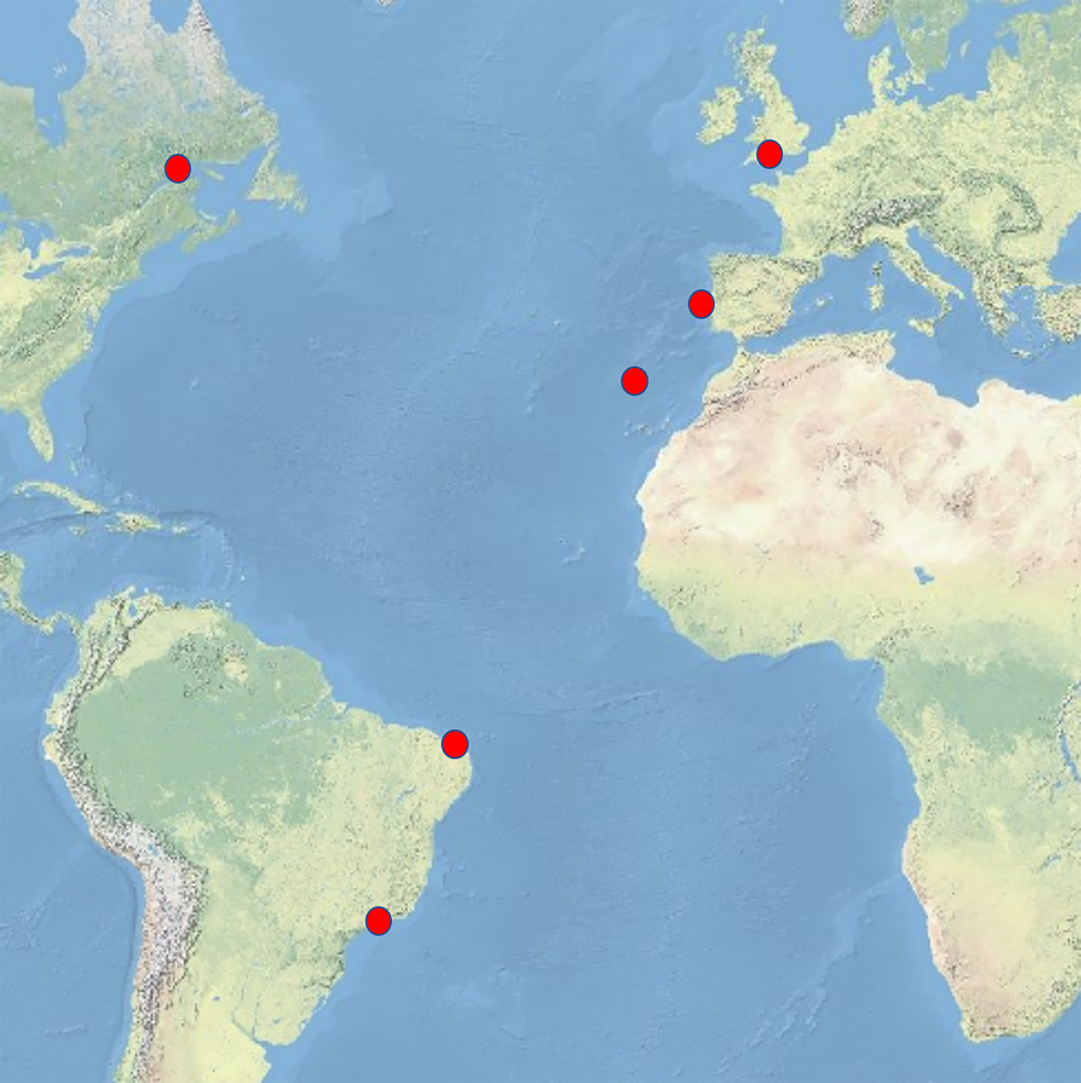
Location of the sampling sites. Red dots mark the location of the sampling sites.

Sampling took place always in summer (2013–2015), during spring tides. This time of the year was chosen, to ensure comparability since it is when biodiversity and species abundance is highest in the intertidal rock pools, compared to other seasons (personal observation). Six ecoregions were sampled: Gulf of Saint Lawrence–Canada, Celtic Sea–United Kingdom, South European Atlantic shelf–Portugal, Madeira Island–Portugal, Northeastern Brazil–Brazil, Southeastern Brazil–Brazil (Fig 4). Two sites were chosen in Canada (Gulf of St. Lawrence, site A—Pointe-au-Père– 48°29’33.0”N 68°29’33.0”W, site B–Sainte-Flavie—48°36'43.0"N 68°13'44.3"W), United Kingdom (South coast, site A–Mount Baten– 50°21’24”N 4°07’43”W, site B–Wembury– 50°19’00”N 4°04’57”W), Portugal-west coast (Portugal mainland, site A–Cabo Raso—38°42'38.2"N 9°29'09"W and site B–Raio Verde—39°17'11.4"N 9°20'23"W), Portugal-Madeira (Madeira Island, northeast Atlantic, site A–Caniço– 32°38’44.4”N 16°49’26.5”W, site B–Porto da Cruz– 32°46’32.6”N 16°49’33.5”W), Brazil-São Paulo (southeast coast, site A–São Sebastião—23°49'26"S 45°25'38"W and site B–Ubatuba—23°28'01"S 45°03'36"W) and Brazil-Ceará (Northeast coast, site A–Flecheiras—3°13'04"N 39°15'29"W and site B–Guajirú—3°14'14"N 39°13'44"W). Sites A and B distanced between 6 and 60 km from each other. In these two sites, 2 to 4 beaches were targeted. All sampled intertidal rock pools were located in the lower intertidal and their size range (depth: 0.05 m—0.80 m; surface area: 0.15 m2–33.00 m^2^, as estimated from scaled digital photographs using the software ImageJ) ensured a minimum patch size for the development of benthic assemblages, while still allowing a complete record of all macro-organisms found in each pool. In total, 28 pools were sampled in Canada, 8 in the UK, 32 in Portugal-west coast, 14 in Portugal-Madeira, 18 in Brazil-São Paulo (Brazil-SP) and 16 in Brazil-Ceará (Brazil-CE) (see S1 Table for the main pool characteristics).

Pool height (elevation–vertical distance from the mean sea level) and distance from the coastline was also registered. Substrate cover was registered, as well as water temperature (±0.1°C) and salinity (±1‰). One bottom sediment sample of 50 ml was taken from pools with an area ≤0.5 m^2^, two samples from pools with an area >0.5 m^2^ and ≤ 2 m^2^, and three samples from pools with an area >2 m^2^, whenever the pool presented sediment at the bottom. Three quadrats of 5 cm^2^ of rock pool surface were scrapped. Sediment and scraping samples were preserved in alcohol 70°, with Bengal rose, and taken to the laboratory, where all organisms were identified with a stereomicroscope. Fish, shrimp and crabs were collected with hand-nets. Macroalgae, sponges, cnidarians, polychaetes, molluscs and echinoderms were collected by hand. All macro-organisms present in the pools were identified *in situ*, but samples were taken to the laboratory whenever there were taxonomical issues, requiring more detailed observation. In the latter case, marine organisms were identified with the aid of a stereomicroscope, and when necessary by consulting identification keys and taxonomic experts. Microscopic organisms were not included in the food webs, with the exception of zooplankton and phytoplankton that were included as a group due to low resolution of their predators’ diet.

Highly resolved food webs, depicting who eats whom, were compiled for each pool, based on published information on each species diet (see S1 Text and S2 Text).

### Network structure of food webs

The networks analysed were trophic species versions of the food webs. Trophic species are taxa that have the same set of prey and predators [68]. Using trophic species is a convention in structural network studies of food webs, in order to reduce methodological biases of uneven resolution among food webs [1, 68]. This food web networks consist of nodes connected by unweighted, directed links that represent prey-predator relations. For each food web, 11 basic properties of trophic species food webs were calculated (Table 3). A measure of biodiversity was included: number of trophic species (S). Two standard measures of food-web trophic interaction richness are reported: links per species (L/S), which indicates the mean number of links per node; and connectance (C), where C = L/S^2^. Six properties yielded percentages of types of species in a food web: top (T) (taxa that lack any predators or parasites), intermediate (I), and basal species (B) (taxa that lack any prey items); cannibals (Can); omnivores (Omn) (taxa with food chains of different lengths, where a food chain is a linked path from a non-basal to a basal species); and herbivores plus detritivores (H). Resource count and consumer count were also estimated for each trophic species. These are commonly estimated properties in food web network analyses [1,3].

**Table 3:** Definition of the food web properties calculated.

Food web property	Definition of food web property
S	Number of trophic species
L/S	Links per species
C	Connectance, C = L/S^2^
T	Top species (taxa that lack any predators or parasites)
I	Intermediate species
B	Basal species (taxa that lack any prey items)
Can	Cannibals
Omn	Omnivores (taxa with food chains of different lengths, where a food chain is a linked path from a non-basal to a basal species)
H	Herbivores plus detritivores
Resource count	Count of all species that serve as resources in the food web
Consumer count	Count of all species that serve as consumers in the food web
TL	Mean shortweighted trophic level
Chain	Mean number of links in every possible food chain or sequence of links connecting top species to basal species
Path	Mean shortest path length between species pairs
GenSD	Standard deviation of mean generality, how many prey items a species has
VulSD	Standard deviation of mean vulnerability, how many predators a species has
LinkSD	Normalized standard deviation of links, which estimates links per taxon
Clust	Clustering coefficient, the mean fraction of species pairs connected to the same species that are connected to each other

Seven overall properties of trophic webs structure were also quantified (Table 3): mean shortweighted trophic level (TL), a trophic level measure which gives the most accurate estimate of trophic level based on binary link information [69]; mean number of links in every possible food chain, or sequence of links connecting top species to basal species (Chain); characteristic path length (Path), the mean shortest path length between species pairs; standard deviation of mean generality (GenSD) how many prey items a species has; vulnerability (VulSD), how many predators a species has; normalized standard deviation of links (LinkSD), which estimates links per taxon; and clustering coefficient (Clust), the mean fraction of species pairs connected to the same species that are connected to each other [70–73]. The software Network3D [57, 58] was used for all calculations. The ranges of the properties of the food webs examined were compared to those of highly resolved food webs published for other ecosystems (Table 1). The works selected for comparison with the results of the present work are recent works that apply a similar methodology to the one used in the field and lab in the present work and have been used for similar purposes in other recent works dealing with structural food web networks (e.g. [3, 74]). This study includes the same common trophic aggregations conducted in the other published works on food web networks used here for comparative purposes, which also rely on published works on diets. Generally, diet papers aggregate phytoplankton (because it is so difficult to analyse to the species level), zooplankton (for a similar reason); macroalgae (aggregated into large groups: red, brown and green macroalgae); oligochaeta and polychaeta (which are both often digested to a point where species’ identification is not possible). The trophic aggregations are thus imposed by the establishment of feeding links based on published diet studies. This means that the level of resolution of the food webs analysed in the present work is as highly resolved as that previously published by other authors and that direct comparison of the networks is possible.

Linear regressions were calculated for the variation of food web properties with pool area, depth and height (for each location). Only significant correlations with a Pearson coefficient above 0.5 were considered. A significance level of 0.05 was used in all test procedures. All statistical analyses were carried out using the Statistica software (version 12.0, StatSoft Inc., USA).

### The niche model

The capacity of the niche model [1] in predicting food web properties was estimated for each intertidal rock pool food web. The niche model has 2 input parameters: the number of trophic species (S) and connectance (C) of the food web. The niche model assigns each species a randomly drawn ‘niche value’ (n_i_) from the interval (1,0). Each species is then limited to consume all prey species within a range of values (r_i_) whose randomly chosen centre (c_i_) is less than the consumer’s niche value. The niche model allows up to half a consumer’s range to include species with higher niche values than the consumer, thus allowing looping (cycles of >1 length (e.g. A eats B, which eats A, or longer like A eats B, which eats C, which eats A) and cannibalism (cycles of length 1 (A eats A).

Additionally, the consumer must feed on all species that fall within its feeding range (r_i_). For each food web, Monte Carlo simulations were used to generate 1000 niche model webs with the same S and C as the empirical web, allowing the estimation of a model mean and standard deviation for each of the network properties. If the normalized error (raw error divided by model SD) between the empirical property and the mean model value for that property falls with ± 1 model SD, the model is considered to have a good fit to the empirical data [1]. The software Network3D [57, 58] was used for all previous calculations. The percentage of niche model errors (taking into account all food web network properties) was estimated for each pool. Then the mean percentage of niche model errors was estimated for each location. A mean percentage of niche model errors <30% was considered a good fit [3]. Differences in the percentage of niche model errors among locations were analysed using a 1-way ANOVA, followed by Tukey post-hoc tests. The ANOVA assumptions were previously investigated. Normality was investigated through the Shapiro-Wilk’s test and homoscedasticity through a Levene’s test. A significance level of 0.05 was considered in all test procedures.

## Supporting information

S1 Table General characteristics of the pools surveyed. (DOCX)

S2 Table List of all taxa identified in the pools. (DOCX)

S1 Text References used to establish feeding links between the taxa. (DOCX)

S2 Text References used for the identification of the organisms. (DOCX)
