# Supplementary material for: What's in a tide pool? Just as much food web network complexity as in large open ecosystems
Source: PLoS One. 2018 Jul 5;13(7):e0200066. doi: 10.1371/journal.pone.0200066 (PMC6033428; doi:10.1371/journal.pone.0200066)
Supplement: S1 Table — (DOCX) [file pone.0200066.s001.docx]

**S1 Table. General characteristics of the pools surveyed.**

|  | Nº pools | TºC  (mean±sd) | Salinity  (mean±sd) | Area (m^2^)  range | Depth (m)  (mean±sd) | Distance to sea (m) (mean±sd) | | Height (m) (mean±sd) |
| --- | --- | --- | --- | --- | --- | --- | --- | --- |
|  |  |  |  |  |  |  |  | |
| Canada | 28 | 16.9±3.6 | 26.3±0.9 | 0.19-3.90 | 0.15±0.03 | 11.4±6.9 | 1.4±0.4 | |
| UK | 8 | 21.1±2.2 | 33.0±0.1 | 0.17-5.40 | 0.22±0.01 | 5.0±0.5 | 1.0±0.1 | |
| Portugal-west coast | 32 | 19.6±0.3 | 35.1±0.9 | 0.16-14.70 | 0.30±0.04 | 9.7±3.9 | 1.1±0.6 | |
| Portugal-Madeira | 14 | 21.6±0.6 | 36.6±0.6 | 0.40-13.79 | 0.34±0.10 | 3.9±1.7 | 1.3±0.1 | |
| Brazil - SP | 18 | 29.4±2.4 | 33.9±0.8 | 0.30-32.50 | 0.35±0.30 | 2.1±0.8 | 0.7±0.3 | |
| Brazil - CE | 16 | 32.0±1.6 | 37.0±0.4 | 0.16-18.78 | 0.32±0.02 | 14.7±1.5 | 0.3±0.1 | |
