# Supplementary material for: What's in a tide pool? Just as much food web network complexity as in large open ecosystems
Source: PLoS One. 2018 Jul 5;13(7):e0200066. doi: 10.1371/journal.pone.0200066 (PMC6033428; doi:10.1371/journal.pone.0200066)
Supplement: S2 Table — (DOCX) [file pone.0200066.s002.docx]

**S2 Table. List of all taxa identified in the pools.**

|  | Taxa |
| --- | --- |
| Canada (Gulf St. Lawrence) | Acari |
|  | *Alaria esculenta* |
|  | *Alitta virens* |
|  | *Antithamnion* sp. |
|  | *Ascophyllum nodosum* |
|  | *Aulactinia stella* |
|  | *Balanus crenatus* |
|  | *Cancer irroratus* |
|  | *Chordaria flagelliformis* |
|  | *Clathromorphum circumscriptum* |
|  | *Coilodesme bulligera* |
|  | Complexe fucus |
|  | *Devaleraea ramentacea* |
|  | Diatomophycea |
|  | *Dictyosiphon foeniculaceus* |
|  | *Eteone longa* |
|  | *Eteone* sp. |
|  | *Fabricia stellaris* |
|  | *Gammarus oceanicus* |
|  | *Gayralia oxysperma* |
|  | Harpacticoida |
|  | *Hediste diversicolor* |
|  | *Hildenbrandia rubra* |
|  | *Hildenbrandia rubra* |
|  | Insecta |
|  | *Jaera (Jaera) albifrons* |
|  | *Lithophyllum* sp. |
|  | *Littorina littorea* |
|  | *Littorina obtusata* |
|  | *Littorina saxatilis* |
|  | *Macoma balthica* |
|  | *Monostroma grevillei* |
|  | *Mytilus* sp. |
|  | *Naineris quadricuspida* |
|  | Nematoda |
|  | Oligochaeta |
|  | *Petalonia fascia* |
|  | *Pholoe minuta* |
|  | *Pholoe* sp. |
|  | Phytoplankton |
|  | Polychaeta |
|  | *Polydora* sp. |
|  | *Polydora websteri* |
|  | *Polysiphonia* sp. |
|  | *Ralfsia fungiformis* |
|  | *Ralfsia verrucosa* |
|  | *Rhodomela confervoides* |
|  | *Rhodomela lycopodioides* |
|  | *Saccharina latissima* |
|  | *Scytosiphon lomentaria* |
|  | *Sertulariidae* |
|  | *Skeneopsis planorbis* |
|  | *Strongylocentrotus droebachiensis* |
|  | *Testudinalia testudinalis* |
|  | *Ulvaria obscura* |
|  | *Wildemania miniata* |
|  | Zooplankton |
| UK | *Acanthochitona crinita* |
|  | *Actinia equina* |
|  | *Amphipholis squamata* |
|  | Ampithoidae |
|  | *Anemonia viridis* |
|  | *Ascophyllum nodosum* |
|  | *Asterina gibbosa* |
|  | *Austrominius modestus* |
|  | *Barleeia* sp. |
|  | Bivalvia |
|  | *Cancer pagurus* |
|  | *Carcinus maenas* |
|  | *Chondrus crispus* |
|  | *Chorda filum* |
|  | *Colpomenia peregrina* |
|  | *Corallina officinalis* |
|  | Cumacea |
|  | *Desmarestia aculeata* |
|  | *Dictyota dichotoma* |
|  | *Ectocarpus siliculosus* |
|  | *Fucus serratus* |
|  | *Fucus vesiculosus* |
|  | *Furcellaria lumbricalis* |
|  | Gammaridae |
|  | *Gibbula cineraria* |
|  | *Gibbula umbilicalis* |
|  | *Gobius paganellus* |
|  | Halacaridae |
|  | *Halurus equisetifolius* |
|  | Harparticoida |
|  | Insecta |
|  | *Jania rubens* |
|  | *Lineus ruber* |
|  | *Lithophyllum incrustans* |
|  | *Littorina Littorea* |
|  | *Littorina saxatilis* |
|  | *Lomentaria articulata* |
|  | *Mastocarpus stellatus* |
|  | *Membranipora membranaceae* |
|  | Mysida |
|  | *Mytilus galloprovincialis* |
|  | Nassariidae |
|  | *Nemalion helminthoides* |
|  | Nematoda |
|  | Nemertea |
|  | Nereididae |
|  | *Nucella lapillus* |
|  | *Nymphon gracile* |
|  | Oligochaeta |
|  | *Omalogyra atomus* |
|  | *Ophiocomina nigra* |
|  | *Oshurkovia littoralis* |
|  | Ostracoda |
|  | *Pagurus bernhardus* |
|  | *Palaemon elegans* |
|  | *Palaemon serratus* |
|  | *Palmaria palmata* |
|  | *Patella depressa* |
|  | *Patella ulyssiponensis* |
|  | *Patella vulgata* |
|  | *Phorcus lineatus* |
|  | *Phymatolithon calcareum* |
|  | Phytoplankton |
|  | Polychaeta |
|  | *Polysiphonia* sp. |
|  | *Pomatoschistus minutus* |
|  | *Procerodes littoralis* |
|  | *Rhodomela confervoides* |
|  | Rissoidae |
|  | *Saccharina latissima* |
|  | *Sargassum muticum* |
|  | *Solenocurtus strigilatus* |
|  | Sphaeromatidae |
|  | *Spirorbis spirorbis* |
|  | Stenothoidae |
|  | *Symphodus melops* |
|  | Tanaidacea |
|  | *Taurulus bubalis* |
|  | Tellinidae |
|  | *Tonicella rubra* |
|  | Turritellidae |
|  | *Ulva intestinalis* |
|  | *Ulva lactuca* |
|  | *Ulva linza* |
|  | *Venerupis* sp. |
|  | Zooplankton |
| Portugal (west coast) | *Acanthochitona crinita* |
|  | *Achelia echinata* |
|  | *Actinia* |
|  | *Actinia equina* |
|  | *Actinia fragacea* |
|  | Actinothoe sphyrodeta |
|  | *Aeolidia papillosa* |
|  | Ammotheidae |
|  | Amphilochidae |
|  | *Amphipholis squamata* |
|  | Amphipoda |
|  | *Ampithoe valida* |
|  | Ampithoidae |
|  | *Anemonia sulcata* |
|  | *Angulus tenuis* |
|  | *Anthura* sp. |
|  | *Aplysia punctata* |
|  | *Asparagopsis armata* |
|  | *Asterina gibbosa* |
|  | *Atherina boyeri* |
|  | *Aulactinia verrucosa* |
|  | Balanus sp. |
|  | *Barleeia* sp. |
|  | *Barnea candida* |
|  | *Bifurcaria bifurcata* |
|  | *Botryllus schlosseri* |
|  | Callianassidae |
|  | Calliopidae |
|  | *Calliostoma zizyphinum* |
|  | *Caprella linearis* |
|  | *Caprella* sp. |
|  | *Carcinus maenas* |
|  | Cardiidae |
|  | *Cardium papillosum* |
|  | *Ceramium ciliatum* |
|  | *Ceramium virgatum* |
|  | *Chaetogammarus sp.* |
|  | *Chiton (Rhyssoplax) olivaceus* |
|  | *Chondrus crispus* |
|  | *Chrysallida pellucida* |
|  | Chthamalus sp. |
|  | *Cladophora rupestris* |
|  | *Codium* sp. |
|  | Coleoptera |
|  | *Colpomenia peregrina* |
|  | *Corallina officinalis* |
|  | *Coryphoblennius galerita* |
|  | Cumacea |
|  | *Cymodoce truncata* |
|  | *Cystoseira* |
|  | *Diaphorodoris papillata* |
|  | *Dictyopteris polypodioides* |
|  | *Dictyota dichotoma* |
|  | *Diplodus sargus sargus* |
|  | Egg *Sepia officinalis* |
|  | *Ellisolandia elongata* |
|  | *Endeis spinosa* |
|  | *Epitonium clathrus* |
|  | *Eriphia verrucosa* |
|  | *Eulalia viridis* |
|  | *Felimida purpurea* |
|  | *Fucus vesiculosus* |
|  | Gammaridae |
|  | *Gammarus* sp. |
|  | *Gelidium corneum* |
|  | *Gelidium spinosum* |
|  | *Gibbula umbilicalis* |
|  | *Gobius niger* |
|  | *Gobius paganellus* |
|  | *Gracilariopsis longissima* |
|  | Halacaridae |
|  | Harparticoida |
|  | *Heterosiphonia* sp. |
|  | *Hippolyte varians* |
|  | *Holothuria sp.* |
|  | *Hydrobia* sp. |
|  | *Hypselodoris* sp. |
|  | Idoteidae |
|  | Insecta |
|  | Iphimediidae |
|  | Ischyroceridae |
|  | *Jaera* sp. |
|  | *Jania* sp. |
|  | *Laurencia pinnatifida* |
|  | *Leathesia marina* |
|  | *Lepadogaster lepadogaster* |
|  | *Lepidochitona cinerea* |
|  | *Lepidotrigla* |
|  | *Leptochiton algesirensis* |
|  | Leucothoidae |
|  | *Lipophrys pholis* |
|  | *Lipophrys trigloides* |
|  | *Lithophyllum* |
|  | *Lithophyllum byssoides* |
|  | *Littorina* sp. |
|  | *Liza ramada* |
|  | *Lophozozymus incisus* |
|  | Lysianassidae |
|  | *Maja squinado* |
|  | *Marthasterias glacialis* |
|  | Melitidae |
|  | *Modiolula phaseolina* |
|  | Munnidae |
|  | Muricidae |
|  | *Musculus costulatus* |
|  | Mysida |
|  | *Mytilus galloprovincialis* |
|  | Nassariidae |
|  | *Nassarius reticulatus* |
|  | *Necora puber* |
|  | Nematoda |
|  | Nemertea |
|  | Nereididae |
|  | *Nucella* sp. |
|  | Nymphonidae |
|  | *Ocenebra erinaceus* |
|  | *Octopus vulgaris* |
|  | Oligochaeta |
|  | *Omalogyra atomus* |
|  | *Onchidella celtica* |
|  | Ophiuroidae |
|  | *Pachygrapsus marmoratus* |
|  | *Padina pavonica* |
|  | *Palaemon adspersus* |
|  | *Palaemon elegans* |
|  | *Palaemon longirostris* |
|  | *Palaemon serratus* |
|  | *Palmaria palmata* |
|  | Pantopoda |
|  | *Paracentrotus lividus* |
|  | *Patella depressa* |
|  | *Patella rustica* |
|  | *Patella ulyssiponensis* |
|  | *Patella vulgata* |
|  | Petricolinae |
|  | *Phorcus lineatus* |
|  | Phytoplankton |
|  | *Pirimela denticulata* |
|  | *Polinices* sp. |
|  | Polychaeta |
|  | *Porcellana platycheles* |
|  | *Porphyra* sp. |
|  | *Procedores* sp. |
|  | Pyramidellidae |
|  | *Rhodymenia pseudopalmata* |
|  | Rissoidae |
|  | *Sabellaria alveolata* |
|  | *Saccorhiza polyschides* |
|  | *Sagartia elegans* |
|  | *Sardina pilchardus* |
|  | *Sargassum* sp. |
|  | *Setia ugesae* |
|  | Skeneidae |
|  | *Solenocurtus strigilatus* |
|  | *Sphacelaria cirrosa* |
|  | Sphaeromatidae |
|  | *Spirobranchus* sp. |
|  | *Spirorbis spirorbis* |
|  | Stenothoidae |
|  | Tanaidacea |
|  | *Tricolia pullus* |
|  | Turbellaria |
|  | *Turbonilla lactea* |
|  | Turridae |
|  | *Turritella* sp. |
|  | Turritellidae |
|  | *Turtonia minuta* |
|  | *Ulva lactuca* |
|  | *Venerupis* sp. |
|  | *Xantho pilipes* |
|  | Zooplankton |
| Portugal-Madeira | *Acanthochitona crinita* |
|  | *Acanthonyx lunulatus* |
|  | Ammotheidae |
|  | *Amphipholis squamata* |
|  | Ampithoidae |
|  | *Anemonia sargassensis* |
|  | *Barleeia* sp. |
|  | Batzella inops |
|  | *Caprella* sp. |
|  | Cardiidae |
|  | *Caulerpa webbiana* |
|  | *Ceramium* sp. |
|  | *Cladophora prolifera* |
|  | *Cladostephus spongiosus* |
|  | *Codium adhaerens* |
|  | *Codium* sp. |
|  | *Corallina* sp. |
|  | *Coralliophila meyendorffii* |
|  | *Coryphoblennius galerita* |
|  | *Cymodoce truncata* |
|  | *Cystoseira abies-marina* |
|  | *Dictyopteris polypodioides* |
|  | *Dictyota bartayresiana* |
|  | *Dictyota* sp. |
|  | *Eriphia verrucosa* |
|  | *Eulalia viridis* |
|  | Gammaridae |
|  | *Gibbula pennanti* |
|  | *Gibbula umbilicalis* |
|  | *Gobius* sp. |
|  | *Grapsus adscensionis* |
|  | *Halopteris filicina* |
|  | Harparticoida |
|  | *Hypnea* sp. |
|  | Insecta |
|  | Isopoda |
|  | *Laurencia* sp. |
|  | *Lepadogaster zebrina* |
|  | Leucothoidae |
|  | *Liagora* sp. |
|  | *Lipophrys pholis* |
|  | *Lithophyllum* |
|  | *Liza ramada* |
|  | *Lophozozymus incisus* |
|  | Lysianassidae |
|  | Nematoda |
|  | Oligochaeta |
|  | *Omalogyra atomus* |
|  | Ostracoda |
|  | *Pachygrapsus transversus* |
|  | *Padina pavonica* |
|  | *Palaemon elegans* |
|  | *Palaemon* sp. |
|  | *Parablennius parvicornis* |
|  | *Paracentrotus lividus* |
|  | *Patella ulyssiponensis* |
|  | *Petaloconchus* sp. |
|  | *Phorcus lineatus* |
|  | Phytoplankton |
|  | *Pirimela denticulata* |
|  | Polychaeta |
|  | Priapulida |
|  | Rhodomelaceae |
|  | *Sargassum vulgare* |
|  | Serpulidae |
|  | *Spirobranchus* sp. |
|  | *Spirorbis spirorbis* |
|  | Stenothoidae |
|  | *Stramonita haemastoma* |
|  | *Stypocaulon scoparium* |
|  | Tanaidacea |
|  | *Thalassoma pavo* |
|  | *Tricleocarpa cylindrica* |
|  | *Tricolia* sp. |
|  | Turridae |
|  | *Ulva* sp. |
|  | *Valonia* sp. |
|  | Zooplankton |
| Brazil-SP | *Acanthophora spicifera* |
|  | *Alpheus formosus* |
|  | *Amphiroa* sp. |
|  | *Asteronema breviarticulatum* |
|  | *Bachelotia* sp. |
|  | *Barbatia candida* |
|  | *Bathygobius soporator* |
|  | *Bostrychia* sp. |
|  | *Bryopsis* sp. |
|  | *Bunodosoma caissarum* |
|  | *Bunodosoma cangicum* |
|  | *Callinectes sapidus* |
|  | *Carcinus* sp. |
|  | *Caulerpa racemosa* |
|  | *Cerithium atratum* |
|  | *Chaetomorpha gracilis* |
|  | *Clibanarius vittatus* |
|  | *Codium* sp. |
|  | Colomastigidae |
|  | *Colpomenia peregrina* |
|  | Columbellidae |
|  | Copepoda |
|  | *Crassostrea virginica* |
|  | *Cronius ruber* |
|  | Cumacea |
|  | Cuspidariidae |
|  | *Dictyota dichotoma* |
|  | *Dictyota* sp. |
|  | *Echinometra lucunter* |
|  | *Eriphia gonagra* |
|  | *Eucinostomus melanopterus* |
|  | *Eurypanopeus abreviatus* |
|  | *Fissurella clenchi* |
|  | Gammaridae |
|  | *Gelidium* sp. |
|  | Gnatostnaetroidae |
|  | Harpaticoida |
|  | *Holothuria (Halodeima) grisea* |
|  | *Hymeniacidon heliophila* |
|  | *Hypnea musciformis* |
|  | Idotea |
|  | *Ischnochiton striolatus* |
|  | *Isognomon bicolor* |
|  | Isopoda |
|  | *Jania rubens* |
|  | Lischkeia |
|  | *Lithophyllum* |
|  | *Litopenaeus schmitti* |
|  | *Lottia subrugosa* |
|  | *Malacoctenus delalandii* |
|  | *Megabalanus tintinnabulum* |
|  | Metidae |
|  | *Microphrys bicornutus* |
|  | *Morula nodulosa* |
|  | *Mytilaster solisianus* |
|  | Nematoda |
|  | *Nodilittorina lineolata* |
|  | *Odontesthes argentinensis* |
|  | Oedicerotidae |
|  | *Ophiactis savignyi* |
|  | *Pachygrapsus transversus* |
|  | *Padina* sp. |
|  | *Pagurus* sp. |
|  | *Palaemon northropi* |
|  | *Parablennius marmoreus* |
|  | *Perna perna* |
|  | *Petaloconchus* sp. |
|  | *Phragmatopoma caudata* |
|  | Phytoplankton |
|  | Platlyhelminthes |
|  | Polychaeta |
|  | Potamididae |
|  | *Protopalythoa variabilis* |
|  | *Renilla sp* |
|  | *Rhizoclonium riparium* |
|  | *Sargassum cymosum* |
|  | *Scartella cristata* |
|  | Sebidae |
|  | Serpulidae |
|  | *Solenocurtus strigilatus* |
|  | *Sphacelaria sp* |
|  | *Spirobranchus sp.* |
|  | *Stramonita haemastoma* |
|  | *Strombus* sp. |
|  | Tanaidacea |
|  | *Tegula viridula* |
|  | *Tetraclita stalactifera* |
|  | *Ulva lactuca* |
|  | Zooplankton |
| Brazil-CE | *Abudefduf saxatilis* |
|  | *Acanthophora spicifera* |
|  | *Amphiroa* sp. |
|  | Ampithoidae |
|  | *Anemonia sargassensis* |
|  | *Aplysia dactylomela* |
|  | *Astyris lunata* |
|  | *Aurantilaria aurantiaca* |
|  | *Bathygobius soporator* |
|  | *Bittiolum varium* |
|  | *Boonea jadisi* |
|  | *Bostrychia* sp. |
|  | *Brachidontes* sp. |
|  | *Bryopsis pennata* |
|  | *Bryopsis plumosa* |
|  | *Bryopsis* sp. |
|  | *Callinectes ornatus* |
|  | *Callinectes* sp. |
|  | Calliopidae |
|  | *Caulerpa cupressoides* |
|  | *Caulerpa prolifera* |
|  | *Caulerpa racemosa* |
|  | *Caulerpa scalpelliformis* |
|  | *Caulerpa* sp. |
|  | *Centroceras clavulatum* |
|  | *Cerithium atratum* |
|  | *Chaetomorpha gracilaris* |
|  | *Chondria curvilineata* |
|  | *Chthamalus bisinuatus* |
|  | *Cinachyrella alloclada* |
|  | *Clibanarius antillensis* |
|  | *Codium decorticatum* |
|  | *Columbella mercatoria* |
|  | *Corallina* sp. |
|  | *Cryptonemia crenulata* |
|  | Cumacea |
|  | *Cystodytes dellechiajei* |
|  | *Dictyopteris delicatula* |
|  | *Dictyota menstrualis* |
|  | *Dictyota mertensii* |
|  | *Dictyota* sp. |
|  | *Didemnum granulatum* |
|  | *Diplodonta* sp. |
|  | *Engina turbinella* |
|  | *Epialtus brasiliensis* |
|  | *Epitonium* sp. |
|  | *Eriphia gonagra* |
|  | *Eudistoma vannamei* |
|  | *Eulithidium affine* |
|  | *Fissurella rosea* |
|  | Gammaridae |
|  | *Gelidium pusillum* |
|  | *Gracilaria Birdiae* |
|  | *Gracilaria domingensis* |
|  | *Gracilaria* sp. |
|  | *Haemulon parra* |
|  | *Haliclona* sp. |
|  | Harpaticoida |
|  | *Hippolyte obliquimanus* |
|  | *Hypnea musciformis* |
|  | Idotea |
|  | Idoteidae |
|  | *Isaurus* sp. |
|  | *Ischnochiton striolatus* |
|  | *Ischnoplax pectinata* |
|  | Isopoda |
|  | *Leptopecten* sp. |
|  | *Lithophyllum* sp. |
|  | *Lithopoma phoebium* |
|  | *Lobophora variegata* |
|  | *Lysmata* sp. |
|  | *Mangelia* sp. |
|  | *Menippe nodifrons* |
|  | *Mycale arcuiris* |
|  | *Mycale* sp. |
|  | Nematoda |
|  | *Neogonodactylus* sp. |
|  | *Odostomia* sp. |
|  | Oligochaeta |
|  | *Olivancillaria* sp. |
|  | *Ophiura* sp. |
|  | Ophiuroidea |
|  | Ostracoda |
|  | *Pachygrapsus transversus* |
|  | *Padina gymnospora* |
|  | *Pagurus* sp. |
|  | *Palaemon northropi* |
|  | *Panopeus herbstii* |
|  | *Panopeus* sp. |
|  | *Phallusia nigra* |
|  | Phytoplankton |
|  | *Plocamium brasiliense* |
|  | *Plocamium* sp. |
|  | Polychaeta |
|  | *Polysyncraton amethysteum* |
|  | *Pomacanthus paru* |
|  | *Protopalythoa variabilis* |
|  | *Sabellidae* |
|  | *Sargassum cymosum* |
|  | *Sargassum vulgare* |
|  | *Scartella cristata* |
|  | *Siderastea* sp. |
|  | Sipunculidae |
|  | *Spatoglossum schroederi* |
|  | *Sphacelaria* sp*.* |
|  | *Sphoeroides* sp. |
|  | *Spirorbis* sp. |
|  | Stenothoidae |
|  | *Stramonita haemastoma* |
|  | Talitridae |
|  | Tanaidacea |
|  | *Tedania sp.* |
|  | *Tegula viridula* |
|  | Turbellaria |
|  | *Ulva lactuca* |
|  | *Valonia aegagropila* |
|  | *Zoanthus sociatus* |
|  | Zooplankton |
