# Supplementary material for: What's in a tide pool? Just as much food web network complexity as in large open ecosystems
Source: PLoS One. 2018 Jul 5;13(7):e0200066. doi: 10.1371/journal.pone.0200066 (PMC6033428; doi:10.1371/journal.pone.0200066)
Supplement: S1 Text — (DOCX) [file pone.0200066.s003.docx]

**S1 Text. References used to establish feeding links between the taxa.**

Aarnio K, Bonsdorff E. Seasonal variation in abundance and diet of the sand goby, *Pomatoschistus minutus* (Pallas), in a northern Baltic archipelago. Ophelia. 1993; 37: 19–30.

Abele LG. Comparative species composition and relative abundance of decapod crustaceans in marine habitats of Panama. Mar Biol. 1976; 38: 263-278.

Abele LG, Campanella PG, Salmon M. Natural history and social organization of the semiterrestrial grapsid crab *Pachygrapsus transversus* (Gibbes). J Exp Mar Biol Ecol. 1986; 104: 153-170.

Afli A, Boufahja F, Sadraoui S, Ben Mustapha K, Aïssa P, Mrabet R. Functional organization of the benthic macrofauna in the Bizerte lagoon (SW Mediterranean Sea), semi-enclosed area subject to strong environmental/anthropogenic variations. Cah Biol Mar. 2009; 50: 105-117.

Albano PG, Sabelli B. The molluscan assemblages inhabiting the leaves and rhizomes of a deep water *Posidonia oceanica* settlement in the central Tyrrhenian Sea. Sci Mar. 2012; 76: 721-732.

Albrecht C, von Rintelen T, Sereda S, Riedel F. Evolution of ancient lake bivalves: the Lymnocardiinae (Cardiidae) of the Caspian Sea. Hydrobiologia. 2014; 739: 85-94.

Alexander CG. The paragnaths of some intertidal crustaceans. J Mar Biol Assoc UK. 1988; 68: 581-590.

Allmon WD. Natural history of Turritelline gastropods (Cerithiodea: Turritellidae): A status report. Malacologia. 2011; 54: 159-202.

Amaral V. Environmental constraints on population structure and condition of costal and estuarine crabs (Crustacea: Brachyura). PhD dissertation, Faculty of Sciences of the University of Lisbon, PT. 2008.

Ambrose RF, Nelson BV. Predation by *Octopus vulgaris* in the Mediterranean. Mar Ecol. 1983; 4: 251-261.

Andersen NM, Cheng L. The marine insect Halobates (Heteroptera: Gerridae): biology, adaptations distribution, and phylogeny. Oceanogr Mar Biol: Annu Ver. 2004; 42: 119–180.

Andersson S, Persson M, Moksnes P-O, Baden S. The role of the amphipod *Gammarus locusta* as a grazer on macroalgae in Swedish seagrass meadows. Mar Biol. 2009; 156: 969-981.

Ansell AD. Seasonal Changes in Biochemical Composition of the Bivalve *Abra alba* from the Clyde Sea Area. Mar Biol. 1974; 25: 13-20.

Ansell AD, Comely CA, Robb L. Distribution, movements and diet of macrocrustaceans on a Scottish sandy beach with particular reference to predation on juvenile fishes. Mar Ecol Prog Ser. 1999; 176: 115-130.

Arndt CE, Berge J, Brandt A. Mouthpart-atlas of arctic sympagic amphipods-Trophic niche separation based on mouthpart morphology and feeding ecology. J Crustac Biol. 2005; 25: 401-412.

Arrontes J. Diet, food preference and digestive efficiency in intertidal isopods inhabiting macroalgae. J Exp Mar Biol Ecol. 1990; 139: 231-249.

Ates RML. Fishes that eat sea anemones, a review. J Nat Hist. 1989; 23: 71-79.

Ávila SP. The littoral molluscs (Gastropoda, Bivalvia and Polyplacophora) of São Vicente, Capelas (São Miguel Island, Azores): ecology and biological associations to algae. Sociedad Española of Malacologia. 2003; 21: 11-33.

Azevedo JMN, Simas AMV. Age and growth, reproduction and diet of a sublittoral population of the rock goby *Gobius paganellus* (Teleostei, Gobiidae). Hydrobiologia. 2000; 440: 129-135.

Baeta A, Cabral HN, Marques JC, Pardal MA. Feeding ecology of the green crab *Carcinus maenas* in a temperate estuary, Portugal. Crustaceana. 2006; 79: 1181–1193.

Baldó F, Drake P. A multivariate approach to the feeding habits of small fishes in the Guadalquivir Estuary. J Fish Biol. 2002; 61: 21–32.

Bamber RN, Davis MH. Feeding of echinata Hodge (pycnogonida) on marine algae. J Exp Mar Biol Ecol. 1982; 60: 181-187.

Bartsch I. Global diversity of halacarid mites (Halacaridae: Acari: Arachnida) in freshwater. Hydrobiologia. 2008; 595: 317–322.

Bartulovic V, Glamuzina B, Lucic D, Conides A, Jasprica N, Dulcic J. Recruitment and food composition of juvenile thin-lipped grey mullet, *Liza ramada* (Risso, 1826), in the Neretva River estuary (Eastern Adriatic, Croatia). Acta Adriat. 2007; 48: 25-37.

Batista D, Muricy GRS, Andréa BR, Villaça RC. High intraspecific variation in the diet of the french angelfish *Pomacanthus paru* in the south-western Atlantic. Braz J Oceanogr. 2012; 60: 449-454.

Baums IB, Miller MW, Szmant AM. Ecology of a corallivorous gastropod, *Coralliophila abbreviata*, on two scleractinian hosts. II. Feeding, respiration and growth. Mar Biol. 2003; 142: 1093-1101.

Belegratis MR, Bitis I, Economou-Amilli A, Ott JA. Epiphytic patterns of macroalgal assemblages on Cystoseira species (Fucales, Phaeophyta) in the east coast of Attica (Aegean Sea, Greece). Hydrobiologia. 1999; 412: 67-80.

Bergquist DC, Eckner JT, Urcuyo IA, Cordes EE, Hourdez S, Macko SA, Fisher CR. Using stable isotopes and quantitative community characteristics to determine a local hydrothermal vent food web. Mar Ecol Prog Ser. 2007; 330: 49-65.

Bernárdez C, Freire J, González-Gurriarán E. Feeding of the spider crab *Maja squinado* in rocky subtidal areas of the Ría de Arousa (north-west Spain). J Mar Biol Assoc UK. 2000; 80: 95-102.

Bilgin S, Ozen O, Ates AS. Spatial and temporal variation of *Palaemon adspersus*, *Palaemon elegans*, and *Crangon crangon* (Decapoda: Caridea) in the southern Black Sea. Estuar Coast Shelf Sci. 2008; 79: 671–678.

Bischof K, Krabs G, Wiencke C, Hanelt D. Solar ultraviolet radiation affects the activity of ribulose-1,5-bisphosphate carboxylase-oxygenase and the composition of photosynthetic and xanthophyll cycle pigments in the intertidal green alga *Ulva lactuca* L. Planta. 2002; 215: 502-509.

Biskup S, Bertocci I, Arenas F, Tuya F. Functional responses of juvenile kelps, *Laminaria ochroleuca* and *Saccorhiza polyschides*, to increasing temperatures. Aquat Bot. 2014; 113: 117– 122.

Blackmore G. Field Evidence of Metal Transfer from Invertebrate Prey to an Intertidal Predator, Thais clavigera (Gastropoda: Muricidae). Estuar Coast Shelf Sci. 2000; 51: 127–139.

Blankenship LE, Yayanos AA. Universal primers and PCR of gut contents to study marine invertebrate diets. Mol Ecol. 2005; 14: 891-899.

Blaxter JHS. Experimental rearing of pilchard larvae *Sardina pilchardus*. J Mar Biol Assoc UK. 1969; 49: 557-575.

Blaxter JHS, Hunter JR. The biology of Clupeoid fishes. Adv Mar Biol. 1982; 20: 1-223.

Blazewicz-Paszkowycz M, Ligowski R. Diatoms as food source indicator for some Antarctic Cumacea and Tanaidacea (Crustacea). Antarct Sci. 2002; 14: 11-15.

Bode A, Álvarez-Ossorio MT, Carrera P, Lorenzo J. Reconstruction of trophic pathways between plankton and the North Iberian sardine (*Sardina pilchardus*) using stable isotopes. Sci Mar. 2004; 68: 165-178.

Bonaviri C, Fernández TV, Badalamenti F, Gianguzza P, Lorenzo M, Riggio S. Fish versus starfish predation in controlling sea urchin populations in Mediterranean rocky shores. Mar Ecol Prog Ser. 2009; 382: 129-138.

Bonaviri C, Fernández TV, Fanelli G, Badalamenti F, Gianguzza P. Leading role of the sea urchin *Arbacia lixula* in maintaining the barren state in southwestern Mediterranean. Mar Biol. 2011; 158: 2505-2513.

Borme D, Tirelli V, Palomera I. Feeding habits of European pilchard late larvae in a nursery area in the Adriatic Sea. J Sea Res. 2013; 78: 8–17.

Branco JO, Lunardon-Branco MJ, Verani JR, Schveitzer R, Souto FX, Vale WG. Natural Diet of *Callinectes ornatus* Ordway, 1863 (Decapoda, Portunidae) in the Itapocoroy Inlet, Penha, SC, Brazil. Braz Arch Biol Technol. 2002; 45: 35-40.

Brinkhuis BH. Seasonal Variations in Salt-Marsh Macroalgae Photosynthesis. II. *Fucus vesiculosus* and *Ulva lactuca*. Mar Biol. 1977; 44: 177-186.

Bromley RG. Trace Fossils-Biology, Taphonomy and Applications, Second edition. Springer Science Business Media, B V; 1996. pp. 375.

Bulleri F, Bertocci I, Micheli F. Interplay of encrusting coralline algae and sea urchins in maintaining alternative habitat. Mar Ecol Prog Ser. 2002; 243: 101-109.

Cabello-Pasini A, Aguirre-von-Wobeser E, Figueroa FL. Photoinhibition of photosynthesis in *Macrocystis pyrifera* (Phaeophyceae), *Chondrus crispus* (Rhodophyceae) and *Ulva lactuca* (Chlorophyceae) in outdoor culture systems. J Photochem Photobiol B. 2000; 57: 169–178.

Cadien D, Brusca RC. Anthuridean isopods (Crustacea) of California and the temperate Northeast Pacific. SCAMIT Newsletter. 1993; 12: 1-26.

Caine EA. Feeding mechanisms and possible resource partitioning of the Caprellidae (Crustacea: Amphipoda) from Puget Sound, USA. Mar Biol. 1977; 42: 331-336.

Callaghan TP, Karlson R. Summer dormancy as a refuge from mortality in the freshwater bryozoan *Plumatella emarginata*. Oecologia. 2002; 132: 51–59.

Camus L, Gulliksen B. Antioxidant defense properties of Arctic amphipods: comparison between deep-, sublittoral and surface-water species. Mar Biol. 2005; 146: 355–362.

Cannicci S, Gomei M, Boddi B, Vannini M. Feeding Habits And Natural Diet of the Intertidal Crab *Pachygrapsus marmoratus*: Opportunistic Browser or Selective Feeder? Estuar Coast Shelf Sci. 2002; 54: 983–1001

Cannicci S, Gomei M, Dahdouh-Guebas F, Rorandelli R, Terlizzi A. Influence of seasonal food abundance and quality on the feeding habits of an opportunistic feeder, the intertidal crab *Pachygrapsus marmoratus*. Mar Biol. 2007; 151: 1331-1342.

Carefoot TH. Growth and nutrition of *Aplysia punctata* feeding on a variety of marine algae. J Mar Biol Assoc UK. 1967; 47: 565-589.

Carefoot TH. Feeding, Food Preference, and the Uptake of Food Energy by the Supralittoral Isopod Ligia pallasii. Mar Biol. 1973; 18: 228-236.

Carr WES, Adams CA. Food habits of juvenile marine fishes occupying seagrass beds in the estuarine zone near crystal river, Florida. Trans Am Fish Soc. 1973; 102: 511-540.

Chadderton WL, Ryan PA, Winterbourn MJ. Distribution, ecology, and conservation status of freshwater Idoteidae (Isopoda) in southern New Zealand. J R Soc N Z. 2003; 33:529-548.

Chícharo L, Chícharo A, Gaspar M, Alves F, Regala J. Ecological characterization of dredged and non-dredged bivalve fishing areas off south Portugal. J Mar Biol Assoc UK. 2002; 82: 41-50.

Chícharo MA. Nutritional condition and starvation in *Sardina pilchardus* (L.) larvae off southern Portugal compared with some environmental factors. J Exp Mar Biol Ecol. 1998; 225: 123–137.

Chintiroglou Ch, Koukouras A. The feeding habits of three Mediterranean sea anemone species, *Anemonia viridis* (Forskal), *Actinia equina* (Linnaeus) and *Cereus pedunculatus* (Pennant). Helgol Meeresunters. 1992; 46: 53-68.

Chomsky O, Kamenir Y, Hyams M, Dubinsky Z, Chadwick-Furman NE. Effects of feeding regime on growth rate in the Mediterranean Sea anemone *Actinia equina* (Linnaeus). J Exp Mar Biol Ecol. 2004; 299: 217– 229.

Christensen MS. Trophic relationships in juveniles of three species of sparid fishes in the South African marine littoral. Fish Bull. 1978; 76: 389–401.

Christofoletti RA, Murakami VA, Oliveira DN, Barreto RE, Flores AAV. Foraging by the omnivorous crab *Pachygrapsus transversus* affects the structure of assemblages on sub-tropical rocky shores. Mar Ecol Prog Ser. 2010; 420: 125-134.

Coleman O. Burrowing, Grooming, and Feeding Behaviour of Paraceradocus, an Antarctic Amphipod Genus (Crustacea). Polar Biol. 1989; 10: 43-48.

Coleman RA, Underwood AJ, Benedetti-Cecchi L, Aberg P, Arenas F, Arrontes J, Castro J, Hartnoll RG, Jenkins SR, Paula J, Santina PD, Hawkins SJ. A continental scale evaluation of the role of limpet grazing on rocky shores. Oecologia. 2006; 147: 556–564.

Colin PL. Marine Invertebrates and Plants of the Living Reef. T.F.H. Publications; 1978. pp. 206–210.

Conlan KE, Chess JR. Phylogeny and Ecology of a Kelp-boring amphipod, *Peramphithoe styrotrupetes*, new species (Corophioidea: Ampithoidae). J Crustac Biol. 1992; 12: 410-422.

Contessa L, Bird FL. The impact of bait-pumping on populations of the ghost shrimp *Trypaea australiensis* Dana (Decapoda: Callianassidae) and the sediment environment. J Exp Mar Biol Ecol. 2004; 304: 75-97.

Conway DVP, Coombs SH, Fernández de Puelles ML, Tranter PRG. Feeding of larval sardine, *Sardina pilchardus* (Walbaum), off the north coast of Spain. Bol Inst Esp Oceanogr. 1994; 10: 165-175.

Cook EJ, Kelly MS. Effect of variation in the protein value of the red macroalga *Palmaria palmata* on the feeding, growth and gonad composition of the sea urchins *Psammechinus miliaris* and *Paracentrotus lividus* (Echinodermata). Aquaculture. 2007; 270: 207-217.

Coombs EF, Allen JA. The functional morphology of the feeding appendages and gut of *Hippolyte varians* (Crustacea: Natantia). Zool J Linn Soc. 1978; 64: 261-282.

Corner EDS, Leon YA, Bulbrook RD. Steroid Sulphatase, Arylsulphatase and β-glucuronidase in marine invertebrates. J Mar Biol Assoc UK. 1960; 39: 51-61.

Costa E, Oliveira F, Cancela L. Feeding Ecology of *Nereis diversicolor* (O.F. Müller) (Annelida, Polychaeta) on Estuarine and Lagoon Environments in the Southwest Coast of Portugal. Pan-Am J Aquat Sci. 2006; 1: 114–126.

Costalago D, Navarro J, Álvarez-Calleja I, Palomera I. Ontogenetic and seasonal changes in the feeding habits and trophic levels of two small pelagic fish species from the Mediterranean Sea. Mar Ecol Prog Ser. 2012; 460: 169-181.

Costalago D, Palomera I. Feeding of European pilchard (*Sardina pilchardus*) in the northwestern Mediterranean: from late larvae to adults. Sci Mar. 2014; 78: 41-54.

Costalago D, Palomera I, Tirelli V. Seasonal comparison of the diets of juvenile European anchovy *Engraulis encrasicolus* and sardine *Sardina pilchardus* in the Gulf of Lions. J Sea Res. 2014; 89: 64–72.

Crothers JH. Common topshells: an introduction to the biology of *Osilinus lineatus* with notes on other species in the genus. Field Stud. 2001; 10: 115-160.

Crump RG, Emson RH. Some aspects of the population dynamics of *Asterina gibbosa* (Asteroidea). J Mar Biol Assoc UK. 1978; 58: 451-466.

Cruz-Rivera E, Hay ME. Can quantity replace quality? Food choice, compensatory feeding, and fitness of marine mesograzers. Ecology. 2000; 81: 201–219.

Cruz-Rivera E, Hay ME. Prey nutritional quality interacts with chemical defenses to affect consumer feeing and fitness. Ecol Monogr. 2003; 73: 483-506.

Curtis LA, Hurd LE. On the broad nutritional requirements of the mud snail, Ilyanassa (Nassarius) obsoleta (Say), and its polytrophic role in the food web. J Exp Mar Biol Ecol. 1979; 41: 289-297.

Dance C. Patterns of Activity of the Sea Urchin *Paracentrotus lividus* in the Bay of Port-Cros (Var, France, Mediterranean). Mar Ecol. 1987; 8: 131-142.

Darwall WRT, Costello MJ, Donnelly R, Lysaght S. Implications of life-history
strategies for a new wrasse fishery. J Fish Biol. 1992; 41: 111-123.

Dauby P, Khomsi A, Bouquegneau J. Trophic Relationships within Intertidal Communities of the Brittany Coasts: a Stable Carbon Isotope Analysis. J Coast Res. 1998; 14: 1202-1212.

Dauby P, Scailteur Y, De Broyer C. Trophic diversity within the eastern Weddell Sea amphipod community. Hydrobiologia. 2001; 443: 69-86.

Dauvin J-C, Desroy N. The food web in the lower part of the Seine estuary: a synthesis of existing knowledge. Hydrobiologia. 2005; 540: 13-27.

Davenport J, Moloney TV, Kelly J. Common sea anemones *Actinia equina* are predominantly sessile intertidal scavengers. Mar Ecol Prog Ser. 2011; 430: 147-155.

De Burgh ME, Fankboner PV. A nutritional association between the bull kelp *Nereocystis luetkeana* and its epizooic bryozoan *Membranipora membranacea*. Oikos. 1978; 31: 69-72.

De Jong-Moreau L, Casanova B, Casanova JP. Detailed comparative morphology of the peri-oral structures of the Mysidacea and Euphausiacea (Crustacea): an indicator for the food preference. J Mar Biol Assoc UK. 2001; 81: 235-241.

De los Santos CB, Pérez-Lloréns JL, Vergara JJ. Photosynthesis and growth in macroalgae: linking functional-form and power-scaling approaches. Mar Ecol Prog Ser. 2009; 377: 113-122.

Declerck CH. The evolution of suspension feeding in gastropods. Biol Rev Camb Philos Soc. 1995; 70: 549-569.

DeMaintenon MJ. Phylogenetic analysis of the Columbellidae (Mollusca: Neogastropoda) and the evolution of herbivory from carnivory. Invertebr Biol. 1999; 118: 258 – 288.

Denitto F, Moscatello S, Belmonte G. Occurrence and distribution pattern of *Palaemon* spp. shrimps in a shallow submarine cave environment: a study case in South-eastern Italy. Mar Ecol. 2009; 30: 416-424.

Diannelidis BE, Delivopoulos SG. The Effects of Zinc, Copper and Cadmium on the Fine Structure of *Ceramium ciliatum* (Rhodophyceae, Ceramiales). Mar Environ Res. 1997; 44: 127-134.

Dias M, Silva A, Cabral HN, Vinagre C. Diet of marine fish larvae and juveniles that use rocky intertidal pools at the Portuguese coast. J Appl Ichthyol. 2014; 30: 841–1104.

Dolenec T, Vokal B, Dolenec M. Nitrogen – 15 signals of anthropogenic nutrient loading in *Anemonia sulcata* as a possible indicator of human sewage impacts on marine coastal ecosystems: a case study of Pirovac Bay and the Murter Sea (Central Adriatic). Croat Chem Acta. 2005; 78: 593-600.

Domettila C, Joselin J, Jeeva S. Phytochemical analysis on some south Indian seaweeds. J Chem Pharm Res. 2013; 5: 275-278.

Doulka E, Kehayias G, Chalkia E, Leonardos ID. Feeding strategies of *Atherina boyeri* (Risso 1810) in a freshwater ecosystem. J Appl Ichthyol. 2012; 29: 200-207.

Dubois S, Barillé L, Cognie B. Feeding response of the polychaete *Sabellaria alveolata* (Sabellariidae) to changes in seston concentration. J Exp Mar Biol Ecol. 2009; 376: 94-101.

Duffy JE, Hay ME. Herbivore resistance to seaweed chemical defense: the roles of mobility and predation risk. Ecology. 1994; 75: 1304-1319.

Dworschak PC, Koller H, Abed-Navandi D. Burrow structure, burrowing and feeding behaviour of *Corallianassa longiventris* and *Pestarella tyrrhena* (Crustacea, Thalassinidea, Callianassidae). Mar Biol. 2006; 148: 1369–1382.

Edelman-Furstenberg Y. Ecological Trends Across A Human-Impact Organic Load Gradient Along the Mediterranean Shore: Benthic Macrofaunal Evidence. The Ministry of National Infrastructures, Geological Survey of Israel; 2008. pp. 72.

Edmunds M, Potts GW, Swinfen RC, Waters VL. Defensive behaviour of sea anemones in response to predation by the opisthobranch mollusc *Aeolidia papillosa* (L.). J Mar Biol Assoc UK. 1976; 56: 65-83.

Emson RH. The feeding and consequent role of *Eulalia viridis* O. F. Muller) (Polychaeta) in intertidal communities. J Mar Biol Assoc UK. 1977; 57: 93-6.

Emson RH, Crump RG. Description of a new species of Asterina (Asteroidea), with an account of its ecology. J Mar Biol Assoc UK. 1979; 59: 77-94.

Esquete P, Wilson GDF, Troncoso JS. Ecology and systematics of a new species of Uromunna (Crustacea: Isopoda) from Spanish eelgrass beds. Helgol Mar Res. 2014; 68: 329-339.

Fabri MC, Bargain A, Briand P, Gebruk A, Fouquet Y, Morineaux M, Desbruyères D. The hydrothermal vent community of a new deep-sea field, Ashadze-1, 12°58′N on the Mid-Atlantic Ridge. J Mar Biol Assoc UK. 2011; 91: 1-13.

Fauchald K, Jumars PA. The diet of worms: A study of polychaete feeding guilds. Oceanogr Mar Biol Annu Ver. 1979; 17: 193–284.

Fautin DG, Allen GR. Anemone fishes and their host sea anemones. Tetra-Press, Germany; 1994. pp. 158.

Fenchel T, Kofoed LH, Lappalainen A. Particle Size-Selection of Two Deposit Feeders: the Amphipod *Corophium volutator* and the Prosobranch *Hydrobia ulvae*. Mar Biol. 1975; 30: 119-128.

Fernández C, Boudouresque CF. Phenotypic plasticity of *Paracentrotus lividus* (Echinodermata: Echinoidea) in a lagoonal environment. Mar Ecol. 1997; 152: 145–154.

Fernández IM, González-Quirós R. Analysis of feeding of *Sardina pilchardus* (Walbaum, 1792) larval stages in the central Cantabrian Sea. Sci Mar. 2006; 70: 131-139.

Ferrari I, Chieregato AR. Feeding-habits of juvenile stages of *Sparus auratus* L., *Dicentrarchus labrax* L. and Mugilidae in a brackish embayment of the Po River Delta. Aquaculture. 1981; 25: 243–257.

Figueiredo M, Morato T, Barreiros JP, Afonso P, Santos RS. Feeding ecology of the white seabream, *Diplodus sargus*, and the ballan wrasse, *Labrus bergylta*, in the Azores. Fish Res. 2005; 75: 107–119.

Figueroa FL, Domínguez-González B, Korbee N. Vulnerability and acclimation to increased UVB radiation in three intertidal macroalgae of different morpho-functional groups. Mar Environ Res. 2014; 97: 30-38.

Finicol AT. The Feeding Habits of the Galatheidea. J Mar Biol Assoc UK. 1932; 18: 87-106.

Fish JD, Fish S. Annelida. In: Fish JD (ed.). A Student's Guide to the Seashore. Department of Biological Sciences, The University College of Wales Aberystwyth, London; 1989. pp. 136-180.

Fish JD, Fish S. Arthropoda. In: Fish JD (ed.). A Student's Guide to the Seashore. Department of Biological Sciences, The University College of Wales Aberystwyth, London; 1989. pp. 282-345.

Fish JD, Fish S. Mollusca. In: Fish JD (ed.). A Student's Guide to the Seashore. Department of Biological Sciences, The University College of Wales Aberystwyth, London; 1989. pp. 183-278.

Fish JD, Fish S. Nemertea. In: Fish JD (ed.). A Student's Guide to the Seashore. Department of Biological Sciences, The University College of Wales Aberystwyth, London; 1989. pp. 129-131.

Fish JD, Fish S. Platyhelminthes. In: Fish JD (ed.). A Student's Guide to the Seashore. Department of Biological Sciences, The University College of Wales Aberystwyth, London; 1989. pp. 125-128.

Fish S. Chordata. In: Fish JD (ed.). A Student's Guide to the Seashore. Department of Biological Sciences, The University College of Wales Aberystwyth, London; 1989. pp. 393-427.

Fish S. Echinodermata. In: Fish JD (ed.). A Student's Guide to the Seashore. Department of Biological Sciences, The University College of Wales Aberystwyth, London; 1989. pp. 369-383.

Freire J, González-Gurriarán E. Feeding ecology of the velvet swimming crab *Necora puber* in mussel raft areas of the Ria de Arousa (Galicia, NW Spain). Mar Ecol Prog Ser. 1995; 119: 139-154.

Fretter V. The structure and life history of some minute prosobranchs of rock pools: *Skeneopsis planorbis* (Fabricius), *Omalogyra atomus* (Philippi), *Rissoella diaphana* (Alder) and *Rissoella opalina* (Jeffreys). J Mar Biol Assoc UK. 1948; 27: 597-632.

Fretter V. *Turbonilla elegantissima* (Montagu) a parasitic opisthobranch. J Mar Biol Assoc UK. 1951; 30: 37-47.

Fretter V. Some observations on *Tricolia pullus* (L.) and *Margarites helicinus* (Fabricius). Proc Malacol Soc Lond. 1955; 31: 565-585.

Fretter V, Graham A. The structure and mode of life of the Pyramidellidae, parasitic opisthobranchs. J Mar Biol Assoc UK. 1949; 28: 493-532.

Fretter V, Manly R. Algal associations of *Tricolia pullus*, *Lacuna vincta* and *Cerithiopsis tubercularis* (Gastropoda) with special reference to the settlement of their larvae. J Mar Biol Assoc UK. 1977; 57: 999-1017.

Gamito S, Pires A, Pita C, Erzini K. Food Availability and the Feeding Ecology of Ichthyofauna of a Ria Formosa (South Portugal) Water Reservoir. Estuaries. 2003; 26: 938–948.

Gao K, McKinley KR. Use of macroalgae for marine biomass production and CO2 remediation: a review. J Appl Phycol. 1994; 6: 45-60.

Garstang W. A complete list of the opisthobranchiate Mollusca found at Plymouth; with further observations on their morphology, colours, and natural history. J Mar Biol Assoc UK. 1980; 1: 399-457.

Gaudêncio MJ, Cabral HN. Trophic structure of macrobenthos in the Tagus estuary and adjacent coastal shelf. Hydrobiologia. 2007; 587: 241–251.

Gee JM, Warwick RM, Davey JT, George CL. Field Experiments on the Role of Epibenthic Predators in Determining Prey Densities in an Estuarine Mudflat. Estuar Coast Shelf Sci. 1985; 21: 429-448.

Gibson RN. The Vertical Distribution and Feeding Relationships of Intertidal Fish on the Atlantic Coast of France. J Anim Ecol. 1972; 41: 189-207.

Giere O. Ecology and biology of marine oligochaeta – an inventory rather than another review. Hydrobiologia. 2006; 564: 103–116.

Gisbert E, Cardona L, Castelló F. Resource Partitioning Among Planktivorous Fish Larvae and Fry in a Mediterranean Coastal Lagoon. Estuar Coast Shelf Sci. 1976; 43: 723–735.

Gofas S, Urra J, Salas C. A highly diverse molluscan assemblage associated with eelgrass beds (*Zostera marina* L.) in the Alboran Sea: Micro-habitat preference, feeding guilds and biogeographical distribution. Sci Mar. 2009; 73: 679-700.

Gon O, Ben-Tuvia A. The biology of Boyer’s sand smelt, *Atherina boyeri* Risso, in the Bardawil Lagoon on the Mediterranean coast of Sinai. J Fish Biol. 1983; 22: 537–547.

González-Ortegón E, Cuesta JA, Pascual E, Drake P. Assessment of the interaction between the white shrimp, *Palaemon longirostris*, and the exotic oriental shrimp, *Palaemon macrodactylus*, in a European estuary (SW Spain). Biol Invasions. 2010; 12: 1731-1745.

Good JÁ. Intertidal Aerobic Sandflats as a Habitat for Marine Coleoptera (Carabidae: Heteroceridae, Staphylinidae) in Ireland. Ir Nat J. 1998; 26: 73-80.

Goud J, Gittenberger E, Gittenberger A. Epitoniid parasites (Gastropoda, Caenogastropoda, Epitoniidae) and their host sea anemones (Cnidaria, Actiniaria, Ceriantharia) in the Spermonde archipelago, Sulawesi, Indonesia. Basteria. 2007; 71: 33-56.

Graham A. On the structure and function of the alimentary canal of the limpet. Trans R Soc Edinb. 1932; 57: 287-308.

Graham A. On a ciliary process of food collecting in the gastropod Turritella communis Risso. Proc Zool Soc Lond. 1938; 108: 543–563.

Guerao G, Ribera C. Locomotor activity patterns and feeding habits in the prawn *Palaemon serratus* (Pennant, 1777) (Decapoda, Palaemonidae) in the Alfacs Bay, Ebro Delta, Spain. Crustaceana. 1996; 69: 101-112.

Guerao, G. Feeding habits of the prawns *Processa edulis* and *Palaemon adspersus* (Crustacea, Decapoda, Caridea) in the Alfacs Bay, Ebro Delta (NW Mediterranean). Misc Zool. 1994; 17: 115-122.

Guerra MT, Gaudencio MJ. Aspects of the ecology of *Patella* spp. on the Portuguese coast. Hydrobiologia. 1986; 142: 57-69.

Guerra-García JM, Tierno de Figueroa JM. What do caprellids (Crustacea: Amphipoda) feed on? Mar Biol. 2009; 156: 1881-1890.

Guerra-García JM, Tierno de Figueroa JM, Navarro-Barranco C, Ros M, Sánchez-Moyano JE, Moreira J. Dietary analysis of the marine Amphipoda (Crustacea: Peracarida) from the Iberian Peninsula. J Sea Res. 2013; 85: 508-517.

Haanes H, Gulliksen B. A high local species richness and biodiversity within high-latitude calcareous aggregates of tube-building polychaetes. Biodivers Conserv. 2011; 20: 793-806.

Häder DP, Lebert M, Mercado J, Aguilera J, Salles S, Flores-Moya A, Jiménez C, Figueroa FL. Photosynthetic oxygen production and PAM fluorescence in the brown alga *Padina pavonica* measured in the field under solar radiation. Mar Biol. 1996; 127: 61-66.

Hall SJ, Todd CD. Prey-species selection by the anemone predator *Aeolidia papillosa* (L.): The influence of ingestive conditioning and previous dietary history, and a test for switching behaviour. J Exp Mar Biol Ecol. 1984; 82: 11-33.

Hall SJ, Todd CD, Gordon AD. The influence of ingestive conditioning on the prey species selection in *Aeolidia papillosa* (Mollusca: Nudibranchia). J Anim Ecol. 1982; 51: 907-921.

Hamerlynck O, Cattrijss A. The food of *Pomatoschistus minutus* (Pisces, Gobiidae) in
Belgian coastal waters, and a comparison with the food of its potential competitor P. lozanoi. J Fish Biol. 1993; 44: 753-771.

Hammond RA. The surface of *Priapulus caudatus* (Lamarck, 1816) (Nemathelminthes,
Priapulida). Z Morph Tiere. 1970; 68: 255-268.

Hanelt D, Wiencke C, Nultsch W. Influence of UV radiation on the photosynthesis of Arctic macroalgae in the field. J Photochem Photobiol B. 1997; 38: 40-47.

Harms J, Meyer-Harms B, Dawirs RR, Anger K. Growth and physiology of *Carcinus maenas* (Decapoda, Portunidae) larvae in the field and in laboratory experiments. Mar Ecol Prog Ser. 1994; 108: 107-118.

Harris AC. Cryptic colouration and melanism in the sand-burrowing beetle *Chaerodes trachyscelides* (Coleoptera: Tenebrionidae). J R Soc N Z. 1988; 18: 333-339.

Hawkins SJ, Watson DC, Hill AS, Harding SP, Kyriakides MA, Hutchinson S, Norton TA. A comparison of feeding mechanisms in microphagous herbivorous intertidal prosobranchs in relation to resource partitioning. J Molluscan Stud. 1989; 55: 151-165.

Heaven C, Scrosati R. Feeding preference of *Littorina snails* (Gastropoda) for bleached and photosynthetic tissues of the seaweed *Mazzaella parksii* (Rhodophyta). Hydrobiologia. 2004; 513: 239–243.

Hendler G, Miller JE, Pawson DL, Kier PM. Sea stars, sea urchins, and allies: echinoderms of Florida and the Caribbean. Smithsonian Institution Press. Washington, D.C.; 1995. pp. 390.

Hickman CP, Roberts LS, Keen SL, Larson A, L’Anson H, Eisenhour DJ. Smaller Ecdysozoans. In: Ober WC, Garrison CW (eds.). Integrated Principles of Zoology Fourteenth edition. McGraw-Hill Companies; 2008. pp. 384-386.

Hickman CP, Roberts LS, Keen SL, Larson A, L’Anson H, Eisenhour DJ. Flatworms, Mesozoans, and Ribbon Worms. In: Ober WC, Garrison CW (eds.). Integrated Principles of Zoology, Fourteenth edition. McGraw-Hill Companies, 2008. pp 289-293.

Hickman CP, Roberts LS, Keen SL, Larson A, L’Anson H, Eisenhour DJ. Annelids and Allied Taxa. In: Ober WC, Garrison CW (eds.). Integrated Principles of Zoology, Fourteenth edition. McGraw-Hill Companies, 2008. pp. 362-373.

Hickman CP, Roberts LS, Keen SL, Larson A, L’Anson H, Eisenhour DJ. Sponges and Placozoans. In: Ober WC, Garrison CW (eds.). Integrated Principles of Zoology, Fourteenth edition. McGraw-Hill Companies, 2008. pp. 246-250.

Hickman CP, Roberts LS, Keen SL, Larson A, L’Anson H, Eisenhour DJ. Gnathiferans and Smaller Lophotrochozoans. In: Ober WC, Garrison CW (eds.). Integrated Principles of Zoology, Fourteenth edition. McGraw-Hill Companies, 2008. pp. 313-325.

Hickman CP, Roberts LS, Keen SL, Larson A, L’Anson H, Eisenhour DJ. Chaetognaths, Echinoderms, and Hemichordates. In: Ober WC, Garrison CW (eds.). Integrated Principles of Zoology, Fourteenth edition. McGraw-Hill Companies, 2008. pp. 469-481.

Hickman CP, Roberts LS, Keen SL, Larson A, L’Anson H, Eisenhour DJ. Hexapods. In: Ober WC, Garrison CW (eds.). Integrated Principles of Zoology, Fourteenth edition. McGraw-Hill Companies, 2008. pp. 441-448.

Hickman CP, Roberts LS, Keen SL, Larson A, L’Anson H, Eisenhour DJ. Molluscs. In: Ober WC, Garrison CW (eds.). Integrated Principles of Zoology, Fourteenth edition. McGraw-Hill Companies, 2008. pp. 331-342.

Hickman CP, Roberts LS, Keen SL, Larson A, L’Anson H, Eisenhour DJ. Trilobites, Chelicerates, and Myriapods. In: Ober WC, Garrison CW (eds.). Integrated Principles of Zoology, Fourteenth edition. McGraw-Hill Companies, 2008. pp. 402-403.

Hill AS, Hawkins SJ. Seasonal and spatial variation of epilithic microalgae distribution and abundance and its ingestion by *Patella vulgata* on a moderately exposed rocky shore. J Mar Biol Assoc UK. 1991; 71: 403–423.

Hily C, Jean F. Macrobenthic biodiversity in intertidal habitats of the Iroise biosphere reserve (Brittany, France). J Mar Biol Assoc UK. 1977; 77: 311-323.

Holdich DM, Jones DA. The systematics and ecology of a new genus of sand beach isopod (Sphaeromatidae) from Kenya. J Zool (Lond). 1973; 171: 385-395.

Hopkins TL. Food web of an Antarctic midwater ecosystem. Mar Biol. 1985; 89: 197-212.

Hourigan TF, Stanton FG, Motta PJ, Kelley CD, Carlson B. The feeding ecology of three species of Caribbean angelfishes (family Pomacanthidae). Environ Biol Fishes. 1989; 24: 105-116.

Hunnam P, Brown G. Sublittoral nudibranch Mollusca (sea slugs) in Pembrokeshire waters. Field Stud. 1975; 4: 131-159.

Hylleberg J, Christensen JT. Factors affecting the intraspecific competition and size distribution of the periwinkle *Littorina littorea*. Nat Jutl. 1978; 20: 193-202.

Irving AD, Connell SD, Elsdon TS. Effects of kelp canopies on bleaching an photosynthetic activity of encrusting coralline algae. J Exp Mar Biol Ecol. 2004; 310: 1– 12.

Janas U, Baranska A. What is the diet of *Palaemon elegans* Rathke, 1837 (Crustacea, Decapoda), a non-indigenous species in the Gulf of Gdańsk (southern Baltic Sea)? Oceanologia. 2008; 50: 221-237.

Jenkins SR, Hartnoll RG. Food supply, grazing activity and growth rate in the limpet *Patella vulgata* L.: a comparison between exposed and sheltered shores. J Exp Mar Biol Ecol. 2001; 258: 123–139.

Jephson T, Nyström P, Moksnes P-O, Baden SP. Trophic interactions in *Zostera marina* beds along the Swedish coast. Mar Ecol Prog Ser. 2008; 369: 63-76.

Johnston CS. The Ecological Distribution and Primary Production of Macrophytic Marine Algae in the Eastern Canaries. Int Rev Gesamten Hydrobiol Hydrograph. 1969; 54: 473-490.

Jones MB. The mouthparts of the members of the Jaera albifrons group of species (Crustacea: Isopoda) Mar Biol. 1972; 14: 264–270.

Jones MB, Smaldon G. Aspects of the biology of a population of the cosmopolitan brittlestar *Amphipholis squamata* (Echinodermata) from the Firth of Forth, Scotland. J Nat Hist. 1989; 23: 613–625.

Jöst C, Zauke GP. Trace metal concentrations in Antarctic sea spiders (Pycnogonida, Pantopoda). Mar Pollut Bull. 2008; 56: 1396–1399.

Kennish R. Diet composition influences the fitness of the herbivorous crab, *Grapsus albolineatus*. Oecologia. 1996; 105: 22-29.

Kennish R, Williams GA, Lee SY. Algal seasonality on an exposed rocky shore in Hong Kong and the dietary implications for the herbivorous crab *Grapsus albolineatus*. Mar Biol. 1996; 125: 55-64.

Kilar JA, Lou RB. Ecological and behavioural studies of the decorator crab, *Microphrys bicornutus* Latreille (Decapoda: Brachyura): a test of optimum foraging theory. J Exp Mar Biol Ecol. 1984; 74: 157-167.

Kilar JA, Lou RB. The subtleties of camouflage and dietary preference of the decorator crab, *Microphrys bicornutus* Latreille (Decapoda: Brachyura). J Exp Mar Biol Ecol. 1986; 101: 143-160.

Kim JH, DeWreede RE. Distribution and Feeding Preference of a High Intertidal Littorinid. Bot Mar. 1996; 39: 561-569.

King PE, Wyer D, Jarvis JH. Littoral Pycnogonids of Galway Bay. Ir Nat J. 1971; 17: 78-85.

Koulouri P, Dounas C, Arvanitidis C, Koutsoubas D, Eleftheriou A. Molluscan diversity along a Mediterranean soft bottom sublittoral ecotone. Sci Mar. 2006; 70: 573-583.

Kozaric Z, Petrinec Z, Kuzir S, Gjurcevic E, Bazdaric B. Histochemical Analyses of Digestive Enzymes in the Intestine of Adult Large-Scaled Gurnard (*Lepidotrigla cavillone*, Lacepède, 1801). Anat Histol Embryol. 2011; 40: 314–320.

Kruger LM, Griffiths CL. Sources of nutrition in intertidal sea anemones from the south- western Cape, South Africa. S Afr J Zool. 1996; 31: 110-119.

Kübler JE, Raven JA. Consequences of light limitation for carbon acquisition in three rhodophytes. Mar Ecol Prog Ser. 1994; 110: 203-209.

Kuhlenkamp R, Franklin LA, Lüning K. Effect of solar ultraviolet radiation on growth in the marine macroalga *Dictyota dichotoma* (Phaeophyceae) at Helgoland and its ecological consequences. Helgol Mar Res. 2001; 55: 77-86.

Labropoulou M, Markakis G. Morphological-dietary relationships within two assemblages of marine demersal fishes. Environ Biol Fishes. 1998; 51: 309–319.

Labropoulou M, Papadopoulou-Smith KN. Foraging behaviour patterns of four sympatric demersal fishes. Estuar Coast Shelf Sci. 1999; 49: 99–108.

Laseron CF. Review of the Rissoidae of New South Wales. Rec Aust Mus. 1950; 22: 257-287.

Lawton P, Hughes RN. Foraging behaviour of the crab *Cancer pagurus* feeding on the gastropods *Nucella lapillus* and *Littorina littorea*: comparisons with optimal foraging theory. Mar Ecol Prog Ser. 1985; 27: 143-154.

Leblanc C, Schaal G, Cosse A, Destombe C, Valero M, Riera P, Potin P. Trophic and biotic interactions in *Laminaria digitata* beds: which factors influence the persistence of marine kelp forests in northern Brittany? Cah Biol Mar. 2011; 52: 415-427.

Lebour MV. The Food of Post-Larval Fish. New Series. 1918; 6: 433-469.

Lebour MV. The Food of Plankton Organisms. J Mar Biol Assoc UK. 1922; 12: 644-677.

Lepoint G, Nyssen F, Gobert S, Dauby P, Bouquegneau JM. Relative impact of a seagrass bed and its adjacent epilithic algal community in consumer diets. Mar Biol. 2000; 136: 513-518.

Levinsen H, Turner JT, Nielsen TG, Hansen BW. On the trophic coupling between protists and copepods in arctic marine ecosystems. Mar Ecol Prog Ser. 2000; 204: 65-77.

Linda LS. Diets of the brachyuran crabs *Cancer irroratus*, *C. borealis*, and *Ovalipes ocellatus* in the New York Bight. J Crustac Biol. 1993; 13: 723–735.

Little C, Williams GA, Morritt D, Perrins JM, Stirling P. Foraging behaviour of *Patella vulgata* L. in an Irish sea-lough. J Exp Mar Biol Ecol. 1988; 120: 1-21.

Lochhead JH. The feeding and swimming of Conchoecia (Crustacea, Ostracoda).
Biol Bull (Woods Hole). 1968; 134: 456-464.

Lord JP, Lyczkowskib ER, Wilson WH. Behavior and microhabitat selection of the tortoiseshell limpet *Testudinalia testudinalis* in the northwest Atlantic intertidal zone. J Exp Mar Biol Ecol. 2011; 407: 234–240

Lorenti M, Mariani S. Isopod assemblages in the Straits of Magellan: structural and functional aspects. Polar Biol. 1997; 18: 254-259.

Lubchenco J. Plant species diversity in a marine intertidal community: importance of herbivore food preference and algal competitive abilities. Am Nat. 1978; 112: 23-39.

Lubchenco J. Algal zonation in the New England rocky intertidal community: an experimental analysis. Ecology. 1980; 61: 333-344.

Lubchenco J, Gaines SD. A unified approach to marine plant-herbivore interaction. I. Populations and communities. Annu Rev Ecol Syst. 1981; 12: 405-437.

Lüning K, Freshwater W. Temperature tolerance of Northeast Pacific marine algae. J Phycol. 1998; 24: 310-315.

Macdonald TA, Burd BJ, Macdonald VI Roodselaar A. Taxonomic and feeding guild classification for the marine benthic macroinvertebrates of the Strait of Georgia, British Columbia. Can Tech Rep Fish Aquat Sci. 2010; 2874:62.

Madambashi AM, Christofoletti RA, Pinheiro MAA. Natural diet of the crab *Menippe nodifrons* Stimpson, 1859. (Brachyura, Menippidae) in Paranapua Beach, São Vicente (SP), Brasil. Nauplius. 2005; 13: 77–82.

Mancinelli G. On the trophic ecology of Gammaridea (Crustacea: Amphipoda) in coastal waters: A European-scale analysis of stable isotopes data. Estuar Coast Shelf Sci. 2012; 114: 130-139.

Mantelatto FLM, Christofoletti RA. Natural feeding activity of the crab *Callinectes ornatus* (Portunidae) in Ubatuba Bay (São Paulo, Brazil): influence of season, sex, size and molt stage. Mar Biol. 2001; 138: 585-594.

Marina P, Urra J, Rueda JL, Sala C. Composition and structure of the molluscan assemblage associated with a *Cymodocea nodosa* bed in south-eastern Spain: seasonal and diel variation. Helgol Mar Res. 2012; 66: 585-599.

Marshall BA. Skeneidae, Vitrinellidae and Orbitestellidae (Mollusca: Gastropoda) associated with biogenic substrata from bathyal depths off New Zealand and New South Wales. J Nat Hist. 1988; 22: 949-1004.

Martínez B, Pato LS, Rico JM. Nutrient uptake and growth responses of three intertidal macroalgae with perennial, opportunistic and summer-annual strategies. Aquat Bot. 2012; 96: 14-22.

Mata L, Silva J, Schuenhoff A, Santos R. The effects of light and temperature on the photosynthesis of the *Asparagopsis armata* tetrasporophyte (*Falkenbergia rufolanosa*), cultivated in tanks. Aquaculture. 2006; 252: 12–19.

Matta JL, Chapman DJ. Effects of light, temperature and desiccation on the net emersed productivity of the intertidal macroalga *Colpomenia peregrina* Sauv. (Hamel). J Exp Mar Biol Ecol. 1995; 189: 13-27.

Mazé RA, Domínguez J, Pérez-Cardenal D. Diet of *Lipophrys pholis* (L.) (Teleostei, Blenniidae) in Cantabrian coastal waters (Spain). Acta Oecologica. 1999; 20: 435−448.

McLusky DS. Primary consumers. In: Chapman, Hall (eds.) The Estuarine Ecosystem, Second edition, New York; 1989. pp. 77-92.

Meirelles CA, Matthews CH. Relations between shell size and radula size in marine prosobranchs Mollusca Gastropoda. Thalassas. 2003; 19: 45-53.

Mekhanikova IV. Morphology of mandible and lateralia in six endemic amphipods (Amphipoda, Gammaridae) from lake Baikal, in relation to feeding. Crustaceana. 2010; 83: 865-887.

Michler T, Aguilera J, Hanelt D, Bischof K, Wiencke C. Long-term effects of ultraviolet radiation on growth and photosynthetic performance of polar and cold-temperate macroalgae. Mar Biol. 2002; 140: 1117-1127.

Micu D, Todorova V. Biodiversity of the Western Black Sea. MarBEF Newsletter. 2007; 26-29.

Miller JA. The toxoglossan proboscis: structure and function. J Molluscan Stud. 1989; 55: 167-181.

Milton P. Biology of littoral blenniid fishes on the coast of the South-west England. J Mar Biol Assoc UK. 1983; 63: 223-237.

Monteiro NM, Quinteiras SM, Silva K, Vieira MN, Almada VC. Diet preference reflects the ontogenetic shift in microhabitat use in *Lipophrys pholis*. J Fish Biol. 2005; 67: 102-113.

Moore HB. Algal production and the food requirements of a limpet. Proc Malacol Soc Lond. 1938; 23: 117-118.

Moore PG, Rainbow PS, Vader W. On the feeding and comparative biology of iron in coelenterate-associated gammaridean Amphipoda (Crustacea) from N. Norway. J Exp Mar Biol Ecol. 1994; 178: 205-231.

Moran MJ. Effects of prey density, prey size and predator size on rates of feeding by an intertidal predatory gastropod *Morula marginalba* Blainville (Muricidae), on several species of prey. J Exp Mar Biol Ecol. 1985; 90: 97-105.

Morata T, Falco S, Gadea I, Sospedra J, Rodilla M. Environmental effects of a marine fish farm of gilthead seabream (*Sparus aurata*) in the NW Mediterranean Sea on water column and sediment. Aquacult Res. 2013; 46: 59-74.

Moreira F, Assis CA, Almeida PR, Costa JL, Costa MJ. Trophic Relationships in the Community of the Upper Tagus Estuary (Portugal): a Preliminary Approach. Estuar Coast Shelf Sci. 1992; 34: 617-623.

Moreno T, Castro JJ. Community structure of the juvenile of coastal pelagic fish species in the Canary Islands waters. Sci Mar. 1995; 59: 405-413.

Morote E, Olivar MP, Villate F, Uriarte I. A comparison of anchovy (*Engraulis encrasicolus*) and sardine (*Sardina pilchardus*) larvae feeding in the Northwest Mediterranean: influence of prey availability and ontogeny. ICES J Mar Sci. 2010; 67: 897–908.

Morrisey DJ. Differences in effects of grazing by deposit-feeders *Hydrobia ulvae* (Pennant) (Gastropoda : Prosobranchia) and *Corophium arenarium* Crawford (Amphipoda) on sediment microalgal populations. II. Quantitative effects. J Exp Mar Biol Ecol. 1988; 118: 43-53.

Morton B. Observations on the feeding behavior of *Nassarius clarus* (Gastropoda: Nassariidae) in Shark Bay, Western Australia. Molluscan Res. 2003; 23: 239-249.

Navarro E, Iglesias JIP, Ortega MM, Larretxea X. The Basis for a Functional Response to Variable Food Quantity and Quality in Cockles Cerastoderma edule (Bivalvia, Cardiidae). Physiol Zool. 1994; 67: 468-496.

Naylor E. The diet and feeding mechanism of Idotea. J Mar Biol Assoc UK. 1955; 34: 347-355.

Nechev J, Stefanov K, Popov S. Effect of cobalt ions on lipid and sterol metabolism in the marine invertebrates *Mytilus galloprovincialis* and *Actinia equina*. Comp Biochem Physiol. 2006; 144: 112–118.

Newell R. The role of detritus in the nutrition of two marine deposit feeders, the prosobranch *Hydrobia ulvae* and the bivalve *Macoma balthica*. Proc Zool Soc Lond. 1965; 144: 25-45.

Nicol EAT. The ecology of a salt marsh. J Mar Biol Assoc UK. 1935; 20: 203-261.

Nicotri ME. Factors involved in herbivore food preference. J Exp Mar Biol Ecol. 1980; 42: 13-26.

Nieder J. Seasonal Variation in Feeding Patterns and Food Niche Overlap in the Mediterranean Blennies *Scartella cristata*, *Parablennius pilicornis* and *Lipophrys trigloides* (Pisces: Blenniidae). Mar Ecol. 1997; 18: 227-237.

Nielsen AM, Eriksen NT, Iversen JL, Riisgård HU. Feeding, growth and respiration in the polychaetes *Nereis diversicolor* (facultative filter-feeder) and *N. virens* (omnivorous) – a comparative study. Mar Ecoll Prog Ser. 1995; 125: 149-158.

Nikolioudakis N, Isari S, Pitta P, Somarakis S. Diet of sardine *Sardina pilchardus*: an ‘end-to-end’ field study. Mar Ecol Prog Ser. 2012; 453: 173-188.

Nixon M, Maconnachie E. Drilling *by Octopus vulgaris* (Mollusca: Cephalopoda) in the Mediterranean. J Zool (Lond). 1988; 216: 687-716.

Noël LM-LJ, Hawkins SJ, Jenkins SR, Thompson RC. Grazing dynamics in intertidal rockpools: Connectivity of microhabitats. J Exp Mar Biol Ecol. 2009; 370: 9-17.

Nohrén E, Pihl L, Wennhage H. Spatial patterns in community structure of motile epibenthic fauna in coastal habitats along the Skagerrak – Baltic salinity gradient. Estuar Coast Shelf Sci. 2009; 84: 1–10.

Norman CP, Jones MB. Influence of Depth, Season and Moult Stage on the Diet of the Velvet Swimming Crab *Necora puber* (Brachyura, Portunidae). Estuar Coast Shelf Sci. 1992; 34: 71-83.

Olafsson E, Elmgren R, Papakosta O. Effects of the deposit-feeding benthic bivalve *Macoma balthica* on meiobenthos. Oecologia. 1993; 93: 457-462.

Olafsson EB. Contrasting influences of suspension-feeding and deposit-feeding populations of *Macoma balthica* on infaunal recruitment. Mar Ecoll Progr Ser. 1989; 55: 171–179.

Oldfield E. Observations on the anatomy and mode of life of *Lasaea rubra* (Montagu) and *Turtonia minuta* (Fabricius). Proc Malac Soc. 1955; 226-252.

Oliveira PMC, Costa JL, Chaves ML, Cost MJ, Horta M. New additions to the feeding ecology of *Carcinus maenas* (L., 1758) in a South-western Europe estuary (Portugal). Cah Biol Mar. 2010; 51: 229 – 238.

Orav-Kotta H, Kotta J. Seasonal variations in the grazing of *Gammarus oceanicus*, *Idotea baltica*, and *Palaemon adspersus* on benthic macroalgae. Proc Estonian Acad Sci Biol Ecol. 2003; 52: 141-148.

Öztürk B, Mokievsky VO, Topaloglu B. International Workshop on Black Sea Benthos. In: Turkish Marine Research Foundation, Turkey; 2004. pp. 245.

Padilla Verdín CJ, Carballo JL, Camacho ML. A Qualitative Assessment of Sponge-Feeding Organisms from the Mexican Pacific Coast. The Open Mar Biol Journal. 2010; 4: 39-46.

Pakker H, Beekman CAC, Breeman AM. Efficient photoreactivation of UVBR-induced DNA damage in the sublittoral macroalga *Rhodymenia pseudopalmata* (Rhodophyta). Eur J Phycol. 2000; 35: 109-114.

Pasternak Z, Achituv Y. Feeding behaviour of shallow-water barnacles from the Mediterrannean and Red-Sea. J Crust Biol. 2007; 27: 543-547.

Pavia H, Carr H, Aberg P. Habitat and feeding preferences of crustacean mesoherbivores inhabiting the brown seaweed *Ascophyllum nodosum* (L.) Le Jol. and its epiphytic macroalgae. J Exp Mar Biol Ecol. 1999; 236: 15–32.

Peattie ME, Hoare R. The Sublittoral Ecology of the Menai Strait: II. The Sponge *Halichondria panicea* (Pallas) and its Associated Fauna. Estuar Coast Shelf Sci. 1981; 13: 621-635.

Penney AJ, Griffiths CL. Prey slection and the impacto f the starfish Marthasteris glacialis (L.) and other predators on the *Mussel meridionalis* (Krauss). J Exp Mar Biol Ecol. 1984; 75: 19-36.

Pennings SC, Carefoot TH, Zimmer M, Danko JP, Ziegler A. Feeding preferences of supralittoral isopods and amphipods. Canad J Zool. 2000; 78: 1918–29.

Pentecost A. Calcification and photosynthesis in Corallina officinalis L. using the 14CO_2_ method. Br Phycol J. 1978; 13: 383-390

Pereira PHC, Barros B, Zemoi R, Ferreira BP. Ontogenetic diet changes and food partitioning of *Haemulon* spp. coral reef fishes, with a review of the genus diet. Fish Biol Fisheries. 2015; 25: 245-260.

Persic A, Roche H, Ramade F. Stable carbon and nitrogen isotope quantitative structural assessment of dominant species from the Vaccarès Lagoon trophic web (Camargue Biosphere Reserve, France). Estuar Coast Shelf Sci. 2004; 60: 261-272.

Piedras SRN, Pouey JLOF. Feeding of the silverside (*Odontesthes bonariensis*, Atherinopsidae) in the Mirim and Mangueira lagoons, Rio Grande do Sul, Brazil. Iheringia Sér Zool. 2005; 95: 117-120.

Pihl L. Food selection and consumption of mobile epibenthic fauna in shallow marine areas. Mar Ecol Prog Ser. 1985; 22: 169-179.

Pitacco V, Mavric B, Orlando-Bonaca M, Lipej L. Rocky macrozoobenthos mediolittoral community in the Gulf of Trieste (North Adriatic) along a gradient of hydromorphological modifications. Acta Adriat. 2013; 54: 67-86.

Pitacco V, Orlando-Bonaca M, Mavric B, Popovic A, Lipej L. Mollusc fauna associated with the Cystoseira algal associations in the Gulf of Trieste (Northern Adriatic Sea). Mediterr Mar Sci. 2014; 15: 225-238.

Pleijel F. On feeding of *Pholoe minuta*  (Fabricius, 1780) (Polychaeta: Sigalionidae). Sarsia. 1983; 68: 21-23.

Poore AGB, Hill NA, Sotka EE. Phylogenetic and geographic variation in host breadth and composition by herbivorous amphipods in the family Ampithoidae. Evolution. 2007; 62: 21–38.

Pratt MC. Living where the flow is right: How flow affects feeding in bryozoans. Integr Comp Biol. 2008; 48: 808–822.

Prince JS, LeBlanc WG. Comparative feeding preference of *Strongylocentrotus droebachiensis* (Echinoidea) for the invasive seaweed *Codium fragile* ssp. *tomentosoides* (Chlorophyceae) and four other seaweeds. Mar Biol. 1982; 113: 159-163.

Pugh PJA, King PE. Feeding in intertidal Acari. J Exp Mar Biol Ecol. 1995; 94: 269-280.

Quintano E, Ganzedo U, Díez I, Figueroa FL, Gorostiaga JM. Solar radiation (PAR and UVA) and water temperature in relation to biochemical performance of *Gelidium corneum* (Gelidiales, Rhodophyta) in subtidal bottoms off the Basque coast. J Sea Res. 2013; 83: 47–55.

Rainbow PS. An introduction to the biology of British littoral barnacles. Field Stud. 1984; 6: 1-51.

Ramos S, Ré P, Bordalo AA. New insights into the early life ecology of *Sardina pilchardus* (Walbaum, 1792) in the northern Iberian Atlantic. Sci Mar. 2009; 73: 449-459.

Randall JE. Caribbean reef fishes. Third Edition - revised and enlarged, T F H Publications, Inc Ltd, Hong Kong; 1996. pp. 368.

Rasoanarivo R, Folack J, Champalbert G, Becker B. Relations entre communautés phytoplanctoniques et l’alimentation des larves de *Sardina pilchardus* Ealb. Dans le golfe de Fos (Méditerranée occidentale): influence de la lumière sur l’activité alimentaire des larves. J Exp Mar Biol Ecol. 1991; 151: 83-92.

Rebach S, Ristvey A. Enhancement of the Response of Rock Crabs, *Cancer irroratus*, to Prey Odors following Feeding Experience. Biol Bull. 1999; 197: 361-3667.

Reigada ALD, Negreiros-Fransozo ML. Feeding activity of *Callinectes ornatus* Ordway, 1863 and *Callinectes danae* Smith, 1869 (Crustacea, Brachyura, Portunidae) in Ubatuba, SP, Brazil. Hydrobiologia. 2001; 449: 249-252.

Reynolds LK, Carr LA, Boyer KE. A non-native amphipod consumes eelgrass inflorescences in San Francisco Bay. Mar Ecol Prog Ser. 2012; 451: 107-118.

Rhyne AL, Lin J. Effects of different diets on larval development in a peppermint shrimp (*Lysmata* sp.(Risso)). Aquacult Res. 2004; 35: 1179–1185.

Riera P, Escaravage C, Leroux C. Trophic ecology of the rocky shore community associated with the *Ascophyllum nodosum* zone (Roscoff, France): A δ13C vs δ15N investigation. Estuar Coast Shelf Sci. 2009; 81: 143–148.

Robertson R. Wentletraps (Epitoniidae) feeding on sea anemones and corals. J Molluscan Stud. 1963; 35: 51-63.

Rodil IF, Compton TJ, Lastra M. Geographic variation in sandy beach macrofauna community and functional traits. Estuar Coast Shelf Sci. 2013; 1-9.

Rodrigues LR, Absalão R. Shell colour polymorphism in the chiton *Ischnochiton striolatus* (Gray, 1828) (Mollusca: Polyplacophora) and habitat heterogeneity. Biol J Linn Soc. 2005; 85: 543-548.

Ropes JW. The feeding habitats of the green carb Carcinus maenas (L.). Fish Bull. 1968; 67: 183-203.

Rosecchi E. L’alimentation de *Diplodus annularis*, *Diplodus sargus*, *Diplodus vulgaris* et *Sparus aurata* (Pisces, Sparidae) dans le Golfe de Lion et les lagunes littorales. Rev Trav Inst Peches Marit. 1987; 49: 125–141.

Roth S, Wilson JG. Functional analysis by trophic guilds of macrobenthic community structure in Dublin Bay, Ireland. J Exp Mar Biol Ecol. 1998; 222: 195-217.

Rotramel G. Filter-Feeding by the Marine Boring Isopod, *Sphaeroma quoyanum* H. Milne Edwards, 1840 (Isopoda, Sphaeromatidae). Crustaceana. 1975; 28: 7-10.

Rudman WB. Purpose in pattern: The evolution of colour in Chromodorid nudibranchs. J Molluscan Stud. 1991; 57: 5-21.

Rueda JL, Gofas S, Urra J, Salas C. A highly diverse molluscan assemblage associated with eelgrass beds (*Zostera marina* L.) in the Alboran Sea: Micro-habitat preference, feeding guilds and biogeographical distribution. Sci Mar. 2009; 73: 679-700.

Ruppert EE, Fox RS, Barnesm RD. Invertebrate Zoology: A Functional Evolutionary Approach, 7th edn, Brooks/Cole, Cenage Learning, California, USA. 2004.

Sachidhanandam U, Willan RC, Chou LM. Checklist of the Nudibranchs (Opisthobranchia: Nudibranchia) of the South China sea. Raffles Bull Zool. 2000; 8: 513-537.

Salgado JP, Cabral HN, Costa MJ. Feeding ecology of the gobies Pomatoschistus minutus
(Pallas, 1770) and Pomatoschistus microps (Krøyer, 1838) in the upper Tagus estuary, Portugal. Sci Mar. 2004; 68: 425-434.

Salvarina I, Koutrakis M, Leonardos I. Comparative study of feeding behaviour of five Mugilidae species juveniles from two estuarine systems in the North Aegean Sea. J Mar Biol Assoc UK. 2016; 1:15.

Sangiorgio F. Mediterranean and black sea macrofauna: comparing sampling strategies. Msc thesis, University of Aveiro, PT. 2011.

Santina PD, Sonni C, Sartoni G, Chelazzi G. Food availability and diet composition of three coexisting Mediterranean limpets (*Patella* spp.). Mar Biol. 1993; 116: 87-95.

Sartori AF, Printrakoon C, Mikkelsen PM, Bieler R. Siphonal struture in the Veneridae (Bivalvia: Heterodonta) with an assessment of its phylogenetic application and a review of venerids of the Gulf of Thailand. Raffles Bull Zool. 2008; 18: 103-125.

Sayer MDJ, Gibson RN, Atkinson RJA. Growth, diet and condition of corkwing wrasse and rock cook on the west coast of Scotland. J Fish Biol. 1996; 49: 76-94.

Schaal G, Riera P, Leroux C. Trophic coupling between two adjacent benthic food webs within a man-made intertidal area: A stable isotopes evidence. Estuar Coast Shelf Sci. 2008; 77: 523-534.

Schaal G, Riera P, Leroux C Trophic significance of the kelp *Laminaria digitata* (Lamour.) for the associated food web: a between-sites comparison. Estuar Coast Shelf Sci. 2009; 85: 565–572.

Schaal G, Riera P, Leroux C, Grall J. A seasonal stable isotope survey of the food web associated to a peri-urban rocky shore. Mar Biol. 2010; 157: 283-294.

Schander C. Pyramidellidae (Mollusca, Gastropoda, Heterobranchia) of the Faroe Islands. Sarsia. 1995; 80: 55-65.

Scheibling RE, Anthony SX. Feeding, growth and reproduction of sea urchins (*Strongylocentrotus droebachiensis*) on single and mixed diets of kelp (*Laminaria spp.*) and the invasive alga *Codium fragile* ssp. *tomentosoides*. Mar Biol. 2001; 139: 139-146.

Schembri PJ, Jaccarini V. Some Aspects of the Ecology of the Echiuran Worm Bonellia viridis and Associated Infauna. Mar Biol. 1978; 47: 55-61.

Sebens KP. Recruitment in a sea anemone population: juvenile substrate becomes adult prey. Science. 1981; 213: 785-787.

Seed R. Predator-prey relationships between the mud crab *Panopeus herbstii*, the blue crab, *Callinectes sapidus* and the Atlantic ribbed mussel *Geukensia demissa*, R. Seed. Estuar Coast Mar Sci. 1980; 11: 445-458.

Seitz RD, Knick KE, Westphal M. Diet Selectivity of Juvenile Blue Crabs (*Callinectes sapidus*) in Chesapeake Bay. Integr Comp Biol. 2011; 51: 598-607.

Shaw JA, Macey DJ, Brooker LR. Radula synthesis by three species of iron mineralizing molluscs: productionrate and elemental demand. J Mar Biol Assoc UK. 2008; 88: 597-601.

Silliman BR, Layman CA, Geyer K, Zieman JC. Predation by the Black-clawed Mud Crab, *Panopeus herbstii*, in Mid-Atlantic Salt Marshes: Further Evidence for Top-down Control of Marsh Grass Production. Estuaries. 2004; 27: 188–196.

Silva AC, Hawkins SJ, Clarke KR, Boaventura DM, Thompson RC. Preferential feeding by the crab *Necora puber* on differing sizes of the intertidal limpet *Patella vulgata*. Mar Ecol Prog Ser. 2010; 416: 179-188.

Silva AC, Silva IC, Hawkins SJ, Boaventura DM, Thompson RC. Cheliped morphological variation of the intertidal crab *Eriphia verrucosa* across shores of differing exposure to wave action. J Exp Mar Biol Ecol. 2010; 391: 84–91.

Silva ACF. Predation by crabs on rocky shores in North-east Atlantic. Doctoral thesis, School of Biological Sciences; 2008. pp. 227.

Silva ACF, Brazão S, Hawkins SJ, Thompson RC, Boaventura DM. Abundance, population structure and claw morphology of the semi-terrestrial crab *Pachygrapsus marmoratus* (Fabricius, 1787) on shores of differing wave exposure. Mar Biol. 2009; 156: 2591-2599.

Skajaa K, Ferno A, Lokkeborg S, Haugland EK. Basic movement pattern and chemo oriented search towards baited pots in edible crab (*Cancer pagurus* L.). Hydrobiologia. 1998; 372: 143-153.

Sloan NA. Microhabitat and Resource Utilization in Cryptic Rocky Intertidal Echinoderms at Aldabra Atoll, Seychelles. Mar Biol. 1979; 54: 269-279.

Smale MJ, Buchan PR. Biology of *Octopus vulgaris* Off the East Coast of South Africa. Mar Biol. 1981; 65: 1-12.

Smith AM, Sutherland JE, Kregting L, Farr TJ, Winter DJ. Phylomineralogy of the Coralline red algae: Correlation of skeletal mineralogy with molecular phylogeny. Phytochemistry. 2012; 81: 97–108.

Smith CD. Diet of *Octopus vulgaris* in False Bay, South Africa. Mar Biol. 2003; 143: 1127-1133.

Smyth JC. A study of the benthic diatoms of Loch Sween (Argyll). J Ecol. 1955; 43: 149-171.

Soler-Membrives A, Arango CP, Cuadrado M, Munilla T. Feeding biology of carnivore and detritivore Mediterranean pycnogonids. J Mar Biol Assoc UK. 2013; 93: 635-643.

Southgate T. The Biology of *Barleeia unifasciata* (Gastropoda: Prosobranchia) in red algal turfs in S.W. Ireland. J Mar Biol Assoc UK. 1982; 62: 461-468.

Stapleton KL, Long M, Bird FL. Comparative feeding ecology of two spatially coexisting species of ghost shrimp, *Biffarius arenosus* and *Trypaea australiensis* (Decapoda: Callianassidae). Ophelia. 2001; 55: 141-150.

Stebbins TD. Population dynamics and reproductive biology of the commensal isopod *Colidotea rostrata* (Crustacea: Isopoda: Idoteidae). Mar Biol. 1989; 101: 329-337.

Steneck RS, Watling L. Feeding Capabilities and Limitation of Herbivorous Molluscs: A Functional Group Approach. Mar Biol. 1982; 68: 299-319.

Stergiou KI, Karpouzi VS. Feeding habits and trophic levels of Mediterranean fish. Rev Fish Biol Fish. 2002; 11: 217-254.

Stibor H, Vadstein O, Diehl S, Gelzleichter A, Hansen T, Hantzsche F, Katechakis A, Lippert B, Loseth K, Peters C, Roederer W, Sandow M, Sundt-Hansen L, Olsen Y. Copepods act as a switch between alternative trophic cascades in marine pelagic food webs. Ecol Lett. 2004; 7: 321–328.

Sykes AV, Almansa E, Lorenzo A, Andrade JP. Lipid characterization of both wild and cultured eggs of cuttlefish (*Sepia officinalis* L.) throughout the embryonic development. Aquacult Nutr. 2009; 15: 38-53.

Tagatz ME. Biology of the blue crab, *Callinectes sapidus* Rathbun in the St Johns river, Florida. Fish Bull. 1968; 67: 17–33.

Taylor DL. The nutritional relationship of *Anemonia sulcata* (Pennant) and its dinoflagellate symbiont. J Cell Sci. 1969; 4: 751-762.

Terrats A, Petrakis G, Papaconstantinou C. Feeding habits of *Aspitrigla cuculus* (L., 1758) (red gurnard), *Lepidotrigla cavillone* (Lac., 1802) (large scale gurnard) and *Trigloporus lastoviza* (Brunn., 1768) (rock gurnard) around Cyclades and Dodecanese Islands (E. Mediterranean). Mediterr Mar Sci. 2000; 1: 91-104.

Thomassin BA. Feeding behaviour of the felt-, sponge-, and coral-feeder sea stars, mainly Culcita schmideliana. Helgoländer wiss. Meeresunters. 1976; 28: 51-65.

Thompson RC, Johnson LE, Hawkins SJ. A method for spatial and temporal assessment of gastropod grazing intensity in the field: the use of radula scrapes on wax surfaces. J Exp Mar Biol Ecol. 1997; 218: 63–76.

Tomida L, Lee JT, Barreto E. Stomach fullness modulates prey size choice in the frillfin goby, Bathygobius soporator. Zoology. 2012; 115: 283–288.

Topp W, Ring RA. Adaptations of Coleoptera to the marine environment. II. Observations on rove beetles (Staphylinidae) from rocky shores. Can J Zool. 1988; 66: 2469-2474.

Tsurpalo AP, Kostina EE. Feeding Characteristics of Three Species of Intertidal Sea Anemones of the South Kuril Islands. Russ J Mar Biol. 2003; 29: 31–40.

Tween TC. On the occurrence, ecology and behavior of *Onchidlla celtica* (Gastropoda, Onchidiacea) in the Littoral of Cornwall. PhD thesis, Luton College of Higher Education, University of Bedfordshire, UK, 1987.

Urra J, Ramírez AM, Marina P, Salas C, Gofas S, Rueda JL. Highly diverse molluscan assemblages of *Posidonia oceanica* meadows in northwestern Alboran Sea (W Mediterranean): Seasonal dynamics and environmental drivers. Estuar Coast Shelf Sci. 2013; 117: 136-147.

Vaas KF, Vlasblom AG, Koeijer P. Studies on the black goby (*Gobius niger*, Gobiidae, pisces) in the Veerse Meer, SW Netherlands. Neth J Sea Res. 1975; 9: 56-68.

Vadas RL. Preferential feeding: an optimization strategy in sea urchins. Ecol Monogr. 1977; 47: 337–371.

Vader W. Associations between gammarid and caprellid amphipods and medusa. Sarsia. 1972; 50: 51-56.

Van Tussenbroek BI, Vides LC. Filamentous Algae Dominate a Tropical Reef Community in the Mexican Caribbean: an Unexpected Organisation of Reef Vegetation. Bot Mar. 2000; 43: 547-557.

Vasconcelos M, Mendes T, Fortes W, Pereira R. Feeding and decoration preferences of the epialtidae crab *Acanthonyx scutiforms*. Braz Journal Oceanogr. 2009; 57: 137-143.

Vazquez LM, Sanchez JP, Bayle SJT. Effects of *Caulerpa racemosa* var. cylindracea on prey availability: an experimental approach to predation of amphipods by *Thalassoma pavo* (Labridae). Hydrobiologia. 2010; 654: 147-154.

Velasco EM, Gómez-Cama MC, Hernando JA, Soriguer MC. Trophic relationships in an intertidal rockpool fish assemblage in the gulf of Cádiz (NE Atlantic). J Mar Syst. 2010; 80: 248–252.

Verdiell-Cubedo D, Egea-Serrano A, Oliva-Paterna F, Torralva M. Biología trófica de los juveniles del género Liza (Pisces: Mugilidae) en la laguna costera del Mar Menor (SE Península Ibérica). Limnetica. 2007; 26: 67-73.

Vesey G, Langford TE. The biology of the black goby, *Gobius niger* L. in an English south-coast bay. J Fish Biol. 1985; 27: 417-429.

Viejo RM, Aberg P. Temporal and spatial variation in the density of mobile epifauna and grazing damage on the seaweed *Ascophyllum nodosum*. Mar Biol. 2003; 142: 1229–1241.

Villafranca S, Jiménez M. Mollusc communities associated with the Asian green mussel *Perna viridis* (Mollusca: Bivalvia) and their trophic relations on the north coast of Araya Peninsula, Sucre State, Venezuela. Rev Biol Trop. 2006; 54: 135-146.

Vizzini S, Mazzola A. Stable carbon and nitrogen ratios in the sand smelt from a Mediterranean coastal area: feeding habits and effect of season and size. J Fish Biol. 2002; 60: 1498–1510.

Voss R, Dickmann M, Schmidt JO. Feeding ecology of sprat (*Sprattus sprattus* L.) and sardine (*Sardina pilchardus* W.) larvae in the German Bight, North Sea. Oceanologia. 2009; 51: 117-138.

Warner GF, Woodley JD. Suspension-feeding in the brittle-star *Ophiothrix fragilis*. J Mar Biol Assoc UK. 1975; 55: 199-210.

Watling L. Functional morphology of the amphipod mandible. J Nat Hist. 1993; 27: 837-849.

Watson DC, Norton TA. The habitat and feeding preferences of *Littorina obtusata* (L.) and *L. mariae* Sacchi et Rastelli. J Exp Mar Biol Ecol. 1987; 112: 61–72.

Weitzmann B, Mercader L. First report of cleaning activity of *Lepadogaster candolii* (Gobiesocidae) in the Mediterranean Sea. Cybium. 2012; 36: 487-488.

Wells FE, Lalli CM. Reproduction and Brood Protection in the Caribbean Gastropods *Coralliophila abbreviata* and *Coralliophila Caribaea*. J Molluscan Stud. 1977; 43: 79-87.

White KN, Reimer JD. Commensal Leucothoidae (Crustacea, Amphipoda) of the Ryukyu Archipelago, Japan. Part I: ascidian-dwellers. Zookeys. 2012; 163: 13-55.

Whitefleet-Smith LA, Harding JM. Size selectivity by Atlantic mud crabs *Panopeus herbstii* (Milne Edwards) feeding on ivory barnacles *Balanus eburneus* (Gould). J Shellfish Res. 2014; 33: 25-33.

Wiencke C, Davenport J. Respiration and photosynthesis in the intertidal alga *Cladophora rupestris* (L.) Kütz. under fluctuating salinity regimes. J Exp Mar Biol Ecol. 1987; 114: 183-197.

Wieser W. Adaptations of two intertidal isopods. I. Respiration and feeding in Naesa bidentata (Adams) (Sphaeromatidae). J Mar Biol Assoc UK. 1962; 42: 665-682.

Williams AB. Shrimps, lobsters, and crabs of the Atlantic coast of the eastern United States, Maine to Florida. Smithsonian Institution Press. Washington DC USA; 1984. pp. 550.

Wilson JB, Holme NA, Barrett RL. Population dispersal in the brittle-star *Ophiocomina nigra* (Abildgaard) (Echinodermata: Ophiuroidea). J Mar Biol Assoc UK. 1977; 57: 405-439.

Wilson SK. Diversity in the Diet and Feeding Habits of Blennies. In: Patzner RA, Gonçalves EJ, Hastings PA, Kapoor BG (eds.) The biology of blennies. Enfield, NH: Science Publishers; 2009. pp. 139-162.

Wong MH, Chan TD. The Ecology of the marine rove beetle, *Bryothinusa* spp. (Coleoptera: Staphylinidae) in Hong Kong. Hydrobiologia. 1997; 53: 253-256.

Woodley JD. The behaviour of some amphiurid brittle stars. J Exp Mar Biol Ecol. 1975; 18:29–46

Wyer D, King PE. Feeding in British Littoral Pycnogonids. Estuar Coast Mar Sci. 1974; 2: 177-184.

Zander CD. On the biology and food of small-sized fish from the North and Baltic sea areas. II. Investigation of a shallow stony ground off Mon, Denmark. Ophelia. 1979; 18: 179-190.

Zintzen V, Norro A, Massin C, Mallefet J. Spatial variability of epifaunal communities from artificial habitat: Shipwrecks in the Southern Bight of the North Sea. Estuar Coast Shelf Sci. 2008; 76: 327-344.
