# Supplementary material for: What's in a tide pool? Just as much food web network complexity as in large open ecosystems
Source: PLoS One. 2018 Jul 5;13(7):e0200066. doi: 10.1371/journal.pone.0200066 (PMC6033428; doi:10.1371/journal.pone.0200066)
Supplement: S2 Text — (DOCX) [file pone.0200066.s004.docx]

**S2 Text. References used for the identification of the organisms.**

Abbott RT. American Seashells. Second Edition. New York: Van Nostrand Reinhold. 1974, 541pp.

Amaral ACZ, Rizzo AE, Arruda EP. Manual de identificação dos invertebrados marinhos da Região Sudeste-Sul do Brasil, 2006. Volume 1. São Paulo: EdUSP.

Boltovskoy D, ed. South Atlantic Zooplankton 2. Vols. Backhuys, Leiden, The Netherlands. 1999, 1706 pp.

Bourget, ELes animaux littoraux du Saint-Laurent: guide d’identification. Quebec : Presses Université Laval, 1997, 268 pages.

Cabioc'h J, Floc'h JY, Boudouresque CF, Meinesz A, Verlaque M. Guide des algues des mers d'Europe. Publisher, Delachaux et Niestlé, 1992. Original from, Cornell University. Digitized, Jul 13, 2009.

Chabot R, Rossignol A. Algues et faune du littoral du Saint-Laurent maritime: Guide d'identification. Institut des sciences de la mer de Rimouski, Rimouski; Pêches et Océans Canada (Institut Maurice-Lamontagne), Mont-Joli. 2003, 113 pages.

Gibson R, Hextall B, Rogers A. Photographic Guide to the Sea and Shore Life of Britain and North-west Europe, 2001, Oxford University, Oxford.

Ferreira S. Contributo para o estudo das Macroalgas do Intertidal da ilha da Madeira. Diversidade, Distribuição e Sazonalidade. Dissertação de mestrado, Universidade da Madeira, 2011, 112 p.

Hayward P, Shields TN-SC: Sea shore of Britain and Europe (Collins Pocket Guide). 1996, Harper Collins Publishers, London.

Hayward, PJ, Ryland, JS, eds. Handbook of the Marine Fauna of North-West Europe. Oxford, England, UK: Oxford University Press. 1995, ISBN-10: 0198540558

Melo GAS, Manual de identificação dos Crustacea Decapoda do litoral brasileiro: Anomura, Thalassinidea, Palinuridea e Astacidea. 1. ed. São Paulo: Plêiade. 1999, v. 1, p. 556.

Muricy G, Hadju E, Porifera brasilis: guia de identificação das esponjas marinhas mais comuns do sudeste do Brasil. Rio de Janeiro: Museu Nacional, 2006, 104p.
